# Deletion of Tgm2 suppresses BMP‐mediated hepatocyte‐to‐cholangiocyte metaplasia in ductular reaction

**DOI:** 10.1111/cpr.13646

**Published:** 2024-04-16

**Authors:** Yaqing Chen, Yi Yan, Yujing Li, Liang Zhang, Tingting Luo, Xinlong Zhu, Dan Qin, Ning Chen, Wendong Huang, Xiangmei Chen, Liqiang Wang, Xianmin Zhu, Lisheng Zhang

**Affiliations:** ^1^ College of Veterinary Medicine/College of Biomedicine and Health Huazhong Agricultural University Wuhan China; ^2^ Department of Diabetes Complications and Metabolism Diabetes and Metabolism Research Institute, Beckman Research Institute, City of Hope National Medical Center Duarte California USA; ^3^ Department of Nephrology, First Medical Center of Chinese PLA General Hospital Nephrology Institute of the Chinese People's Liberation Army, State Key Laboratory of Kidney Diseases, National Clinical Research Center for Kidney Diseases, Beijing Key Laboratory of Kidney Disease Research Beijing China; ^4^ Department of Hepatobiliary and Pancreatic Surgery Cancer Hospital of Wuhan University (Hubei Cancer Hospital) Wuhan China

## Abstract

Transglutaminase 2 (Tgm2) plays an essential role in hepatic repair following prolonged toxic injury. During cholestatic liver injury, the intrahepatic cholangiocytes undergo dynamic tissue expansion and remodelling, referred to as ductular reaction (DR), which is crucial for liver regeneration. However, the molecular mechanisms governing the dynamics of active cells in DR are still largely unclear. Here, we generated Tgm2‐knockout mice (Tgm2^−/−^) and Tgm2‐^CreERT2^‐Rosa26‐mTmG flox/flox (Tgm2^CreERT2^‐R26T/G^f/f^) mice and performed a three‐dimensional (3D) collagen gel culture of mouse hepatocytes to demonstrate how Tgm2 signalling is involved in DR to remodel intrahepatic cholangiocytes. Our results showed that the deletion of Tgm2 adversely affected the functionality and maturity of the proliferative cholangiocytes in DR, thus leading to more severe cholestasis during DDC‐induced liver injury. Additionally, Tgm2 hepatocytes played a crucial role in the regulation of DR through metaplasia. We unveiled that Tgm2 regulated H3K4me3Q5ser via serotonin to promote BMP signalling activation to participate in DR. Besides, we revealed that the activation or inhibition of BMP signalling could promote or suppress the development and maturation of cholangiocytes in DDC‐induced DR. Furthermore, our 3D collagen gel culture assay indicated that Tgm2 was vital for the development of cholangiocytes in vitro. Our results uncovered a considerable role of BMP signalling in controlling metaplasia of Tgm2 hepatocytes in DR and revealed the phenotypic plasticity of mature hepatocytes.

## Graphical abstract

Tgm2 regulates BMP signalling activation to mediate hepatocyte metaplasia, thereby restoring liver injury. The Tgm2‐BMP signalling axis plays a key role in the structure and function of BECs during ductular reaction.

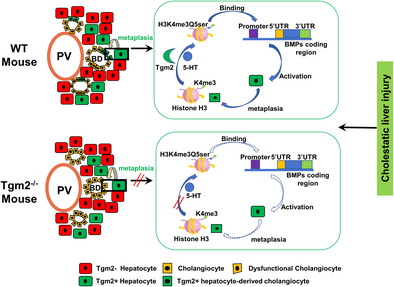

## 1 INTRODUCTION

The hepatic epithelial tissue, composed of hepatocytes and cholangiocytes, is normally inactive under physiological conditions, but exhibits vigorous proliferation and adaptability in response to different injuries, thus contributing to the liver's extraordinary regenerative ability.^1^, ^2^ Upon cholestatic liver injury, the liver parenchyma experiences the ectopic emergence and expansion of atypical biliary epithelial cells (BECs), commonly referred to as ductular reaction (DR), is closely linked to the development of various liver diseases in human patients.^3^ Notably, DR is not only an appealing model for researching the mechanism of biliary remodelling, but also a related regenerative response of the liver to resist various injuries.^4^ Multiple lines of evidence utilizing knockout mice for signalling pathway regulation, including Klf5,^5^ HGF/c‐Met,^6^ TWEAK,^7^ and FGF7,^8^ have universally indicated that inhibition or elimination of DR results in aggravated liver damage and impairments in liver regeneration, whereby revealing that DR has a pro‐regenerative effect on hepatic pathophysiology.^5^, ^6^, ^7^, ^8^ The sources of proliferative BECs in DR can be cholangiocytes, hepatic progenitor cells, and/or hepatocytes, which collaborate to promote the occurrence of DR.^3^ Accordingly, multiple signals and mechanisms have been identified in ductular reaction derived from cholangiocytes or hepatic progenitor cells.^5^, ^9^ Conversely, hepatocyte‐derived DR and its mechanisms and signals for liver injury repair remain poorly unclear. Hepatocytes have the ability to undergo biliary differentiation, leading to the formation of reactive ducts. This phenomenon has been observed in both humans and animals with cholestatic liver injury.^10^ Most studies have demonstrated that hepatocyte‐to‐cholangiocyte metaplasia forms reactive ductules and proliferates around portal vein to repair damaged livers, but these ductule cells tend to revert back into hepatocytes once the liver damage has been repaired.^11^, ^12^, ^13^, ^14^ Mature hepatocytes are suggested to possess the ability to bile ductular metaplasia, but whether and how hepatocellular metaplasia contributes to DR in chronic liver diseases have not been elucidated.

Transglutaminase 2 (Tgm2), a member of the transglutaminase family, is a versatile enzyme mainly localized to the cytoplasm and cell membrane and catalyses Ca2^+^‐dependent protein modification as well as contributes to cell proliferation, differentiation, and apoptosis.^15^, ^16^, ^17^ Strnad et al. reported that Tgm2^−/−^ mice fed with DDC exhibited a higher incidence of gallstones and jaundice than DDC‐fed WT mice.^18^ Nardacci et al. indicated that deficiency of Tgm2 in mice causes more severe hepatobiliary damage in CCl4.^19^ These data indicate that Tgm2 is partly responsible for maintaining tissue stability and participating in liver injury. Tgm2 participates in a variety of diseases caused by the inflammatory process, including wound healing and tissue fibrosis.^20^ Fibrosis, next to inflammation, is the most frequent symptom in chronic cholestatic liver diseases, such as primary biliary cholangitis (PBC) and primary sclerosing cholangitis (PSC).^21^ The multifunctionality of Tgm2 is a potential pharmacological target for the treatment of many fibroproliferative conditions.^22^, ^23^ In this study, we aimed to investigate the novel function and role of Tgm2 in liver injury. Therefore, we first used lineage‐tracing mice to demonstrate that Tgm2 was highly expressed in periportal hepatocytes but not expressed in biliary cells under physiologic conditions and knockout Tgm2 led to more severe cholestasis and fibrosis in DDC‐induced injury and affected the development of BECs in DR.

## 2 RESULTS

### 2.1 The deletion of Tgm2 significantly impacts the structure and function of proliferated BECs during DR

Tgm2 is an acyltransferase that is dependent on calcium and is involved in numerous biological processes.^16^ To investigate the influence of Tgm2 on cholestatic liver injury, we created Tgm2^−/−^ mice through the targeted disruption of Tgm2, resulting in the absence of Tgm2 expression upon the deletion of exons 2–4 of the Tgm2 gene (Figure S1A–D), thereby confirming successful knockout. Under untreated conditions, Tgm2‐knockout mice exhibit normal development of cholangiocytes without discernible defects or damage (Figure S1E–G), and these results are consistent with previous studies.^17^

**FIGURE 1:**
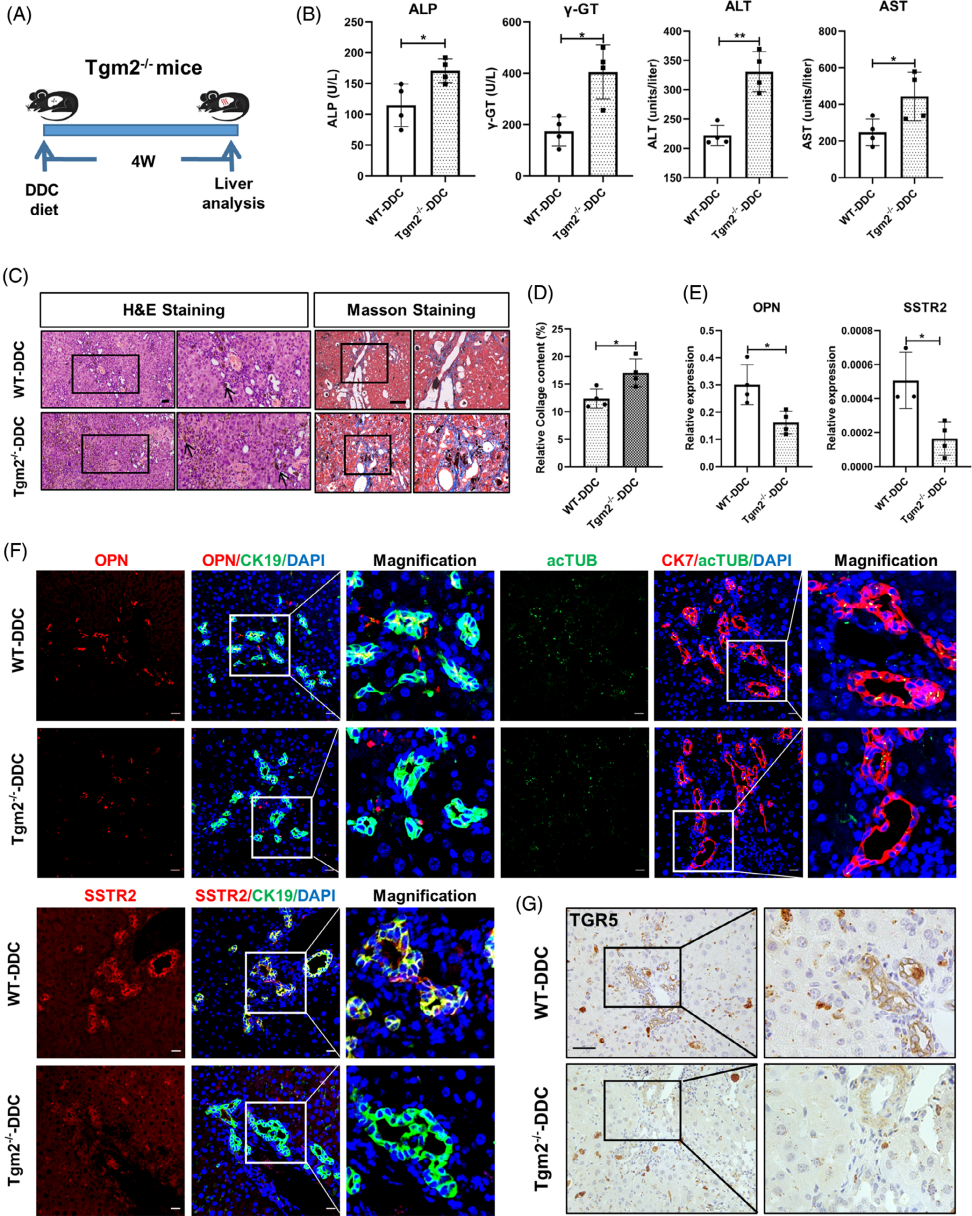
The deletion of Tgm2 has a significant impact on the structure and function of proliferated cholangiocytes during DR. (A) Schematic diagram of WT and Tgm2^−/−^ mouse model after 4‐week DDC injury. (B) Plasma ALP, γ‐GT, ALT, and AST were measured in WT and Tgm2^−/−^ mice after 4‐week DDC injury (*n* = 4/group). (C) H&E staining and Masson's staining in WT and Tgm2^−/−^ mice (*n* = 4/group). Scale bar, 50 μM. Black arrow indicated porphyrin. (D) Quantification of the percentage of collagen‐positive staining areas in Masson's staining (*n* = 4/group). (E) Hepatic expression levels of OPN and SSTR2 were determined in WT and Tgm2^−/−^ mice (*n* ≥ 3/group). (F) Co‐staining of OPN and SSTR2 with the cholangiocyte marker CK19 and co‐staining of acTUB with the cholangiocyte marker CK7 were observed after 4‐week DDC injury (*n* = 4/group). Scale bar, 20 μM. (G) Immunohistochemical staining of TGR5 was observed in WT and Tgm2^−/−^ mice after 4‐week DDC injury (*n* = 4/group). Scale bar, 50 μM. Comparisons between two groups were performed using two‐tailed Student's *t*‐test. *p < 0.05, **p < 0.01, ***p < 0.001, and ****p < 0.0001 represent four different levels of significant difference, respectively.

We then utilized a well‐established mouse model of chronic cholestatic liver injury induced by DDC administration^24^ to investigate the impact of Tgm2. This chronic cholestatic injury model was established in both wild‐type (WT) and Tgm2^−/−^ mice (Figure 1A). Tgm2^−/−^ mice exhibited aggravated DDC‐induced cholestasis, as evidenced by elevated levels of cholestatic markers in the plasma, such as alkaline phosphatase (ALP) and gamma‐glutamyltransferase (γ‐GT). Additionally, the Tgm2^−/−^ mice displayed higher plasma levels of alanine aminotransferase (ALT) and aspartate aminotransferase (AST) compared to the WT mice, indicating hepatocyte injury (Figure 1B). H&E showed more porphyrin in Tgm2^−/−^ group compared to WT group, and Masson's staining demonstrated increased liver fibrosis in Tgm2^−/−^ mice (Figure 1C,D). These findings collectively indicate that the deletion of Tgm2 leads to more severe cholestasis in DDC‐induced injury.

Significant DR with cholangiocyte expansion was observed in DDC‐induced cholestatic liver injury (Figure 1C). The integrity and functionality of cholangiocytes are essential for bile formation, modification, and transportation.^25^ Hence, we assessed the expression of structural and functional markers of ductular cells, including osteopontin (OPN) and somatostatin receptor 2 (SSTR2), and observed reduced expression of these genes in Tgm2^−/−^ mice (Figure 1E). Immunofluorescence staining of mouse liver sections revealed a significant reduction in CK19^+^OPN^+^, CK19^+^SSTR2^+^, and CK7^+^acTUB^+^ cholangiocytes in Tgm2^−/−^ mice, and immunohistochemical staining showed a remarkable decrease in TGR5^+^ (Takeda G protein‐coupled receptor‐5, also known as GPBAR1) BECs in Tgm2^−/−^ mice. These results indicate that the absence of Tgm2 hinders the acquisition of characteristic features of normal biliary cells and restricts the functionality and maturation of proliferated BECs (Figure 1F,G and Figure S2A,B). Besides, we investigated whether hepatic progenitor cells contribute to this process in DR. We examined the expression of CK19^+^SOX9^+^ cells and CK19^+^EpCAM^+^ cells. Our findings revealed no significant difference in WT and Tgm2^−/−^ mice, indicating that SOX9 and EpCAM hepatic progenitor cells may not be involved in this process (Figure S2C,D).

To further validate the observed phenotype, we also employed a 2‐week DDC model in both WT and Tgm2^−/−^ mice, yielding similar results (Figure S2E–H). In conclusion, our findings demonstrate that the deletion of Tgm2 affects the development and function of proliferated BECs during DR, resulting in more severe cholestasis.

### 2.2 Hepatocytes expressing Tgm2 play a crucial role in the regulation of DR through metaplasia

**FIGURE 2:**
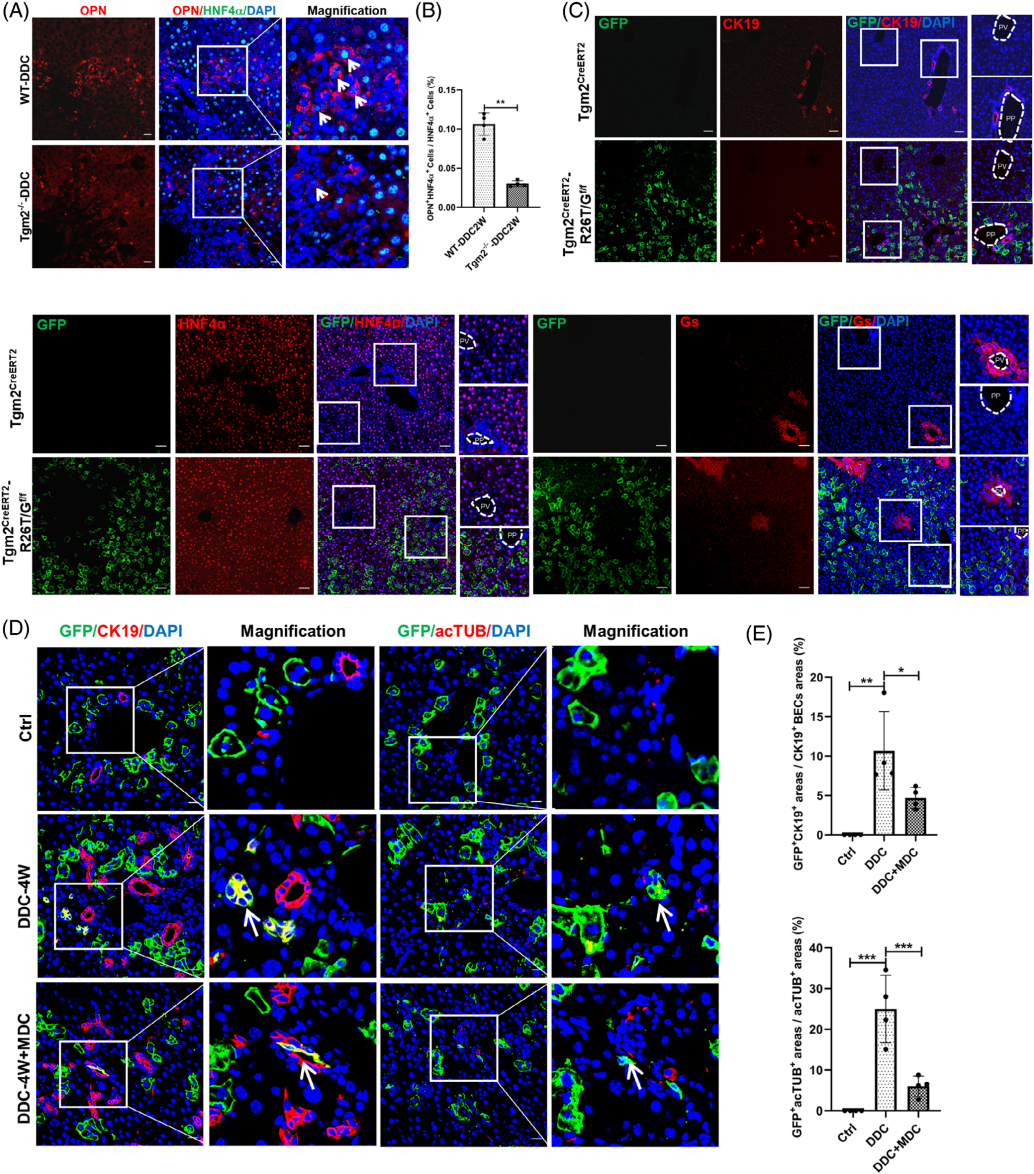
Hepatocytes expressing Tgm2 play a crucial role in the regulation of DR through metaplasia. (A) Two‐week DDC liver injury led to an intermediate phenotype characterized by co‐expression of BEC marker OPN and hepatocyte marker HNF4α (*n* = 4/group). White arrows denoted co‐stained cells. Scale bar, 20 μM. (B) The percentage of OPN^+^HNF4α^+^ cells were determined in HNF4α^+^ hepatocytes (*n* = 4/group). (C) Co‐staining of the CK19, HNF4α, and Gs with the GFP lineage label was observed in Tgm2‐CreERT2 and Tgm2^CreERT2^‐R26T/G^f/f^ mouse livers (*n* = 3/group). Scale bar, 10 μM. (D) Robust staining of the cholangiocyte marker CK19 and primary cilia marker acTUB was observed in GFP^+^ cells after 4‐week DDC injury (*n* = 3/group). White arrows denoted co‐stained cells. Scale bar, 20 μM. (E) The percentage of marker^+^ cells' (i.e., cells that stained positive for CK19 or acTUB) fluorescence intensity was determined in GFP lineage label by fluorescence colocalization analysis (*n* = 4/group). Comparisons between two groups were performed using two‐tailed Student's *t*‐test. *p < 0.05, **p < 0.01, ***p < 0.001, and ****p < 0.0001 represent four different levels of significant difference, respectively.

In the process of DR, it is believed that cells with transitional phenotypes arise from the proliferation and differentiation of hepatic progenitor cells or through metaplasia of mature hepatocytes and BECs.^26^ Human cholestatic liver diseases exhibit a wide range of hepatocyte–BEC transitional phenotypes.^27^ Interestingly, we observed a significant presence of HNF4α^+^OPN^+^ biphenotypic cells in WT mice, while the number of these cells was considerably reduced in Tgm2^−/−^ mice following a 2‐week DDC‐induced injury (Figure 2A,B). To further investigate the role of Tgm2 in the liver, we generated Tgm2^CreERT2^‐R26T/G^f/f^ mice to track the fate of Tgm2^+^ cells. The schematic diagram depicts the Tgm2‐^CreERT2^ knock‐in strategy and tamoxifen‐induced expression of the Tgm2‐reporter GFP gene (Figure S3A,B). A single dose of tamoxifen was administered to mice prior to the DDC diet. Following tamoxifen treatment, Tgm2^+^ cells and their progeny were marked with GFP. Importantly, no GFP^+^ cells were observed after administration of corn oil or the DDC diet alone (Figure S3C). Lineage‐tracing experiments revealed that GFP^+^ cells expressed the hepatocyte marker HNF4α but not the BEC marker CK19, which was abundantly expressed in the periportal area but less expressed in the perivenous area (Figure 2C). Furthermore, none of the labelled GFP^+^ cells expressed markers of hepatic stellate cells (HSCs), such as Col1a1, desmin, or αSMA (Figure S3D). Overall, these results demonstrate that Tgm2 is predominantly expressed in periportal hepatocytes.

During biliary injury‐induced DR, both cholangiocyte self‐proliferation and hepatocyte metaplasia contribute to this process. Therefore, we examined whether Tgm2^+^ hepatocytes undergo metaplasia and transform into cholangiocytes following DDC‐induced liver injury. We treated the mice with the Tgm2 inhibitor dansylcadaverine (MDC)^28^ during DDC injury. After tamoxifen administration, mice were fed with DDC and administered MDC, and liver tissues were collected after 4 weeks. Our results showed that MDC effectively inhibited the expression of Tgm2 (Figure S3E). Masson's staining illustrated more severe liver fibrosis was observed in DDC and MDC group compared to DDC only group (Figure S3F,G). GFP staining demonstrated CK19 and acTUB were expressed in GFP^+^ BECs. However, the number of GFP^+^CK19^+^ cells and GFP^+^acTUB^+^ cells was dramatically decreased when MDC was added to inhibit Tgm2 expression (Figure 2D,E). In addition, the expression of OPN and EpCAM was significantly increased following DDC injury, while OPN and EpCAM expression decreased after treatment with TGM2 inhibitor (Figure S3H). In summary, our findings provide support for the involvement of Tgm2^+^ hepatocytes in DR through metaplasia during cholestatic liver injury.

### 2.3 Tgm2 regulates H3K4me3Q5ser via serotonin, thus activating BMP signalling to regulate DR

**FIGURE 3:**
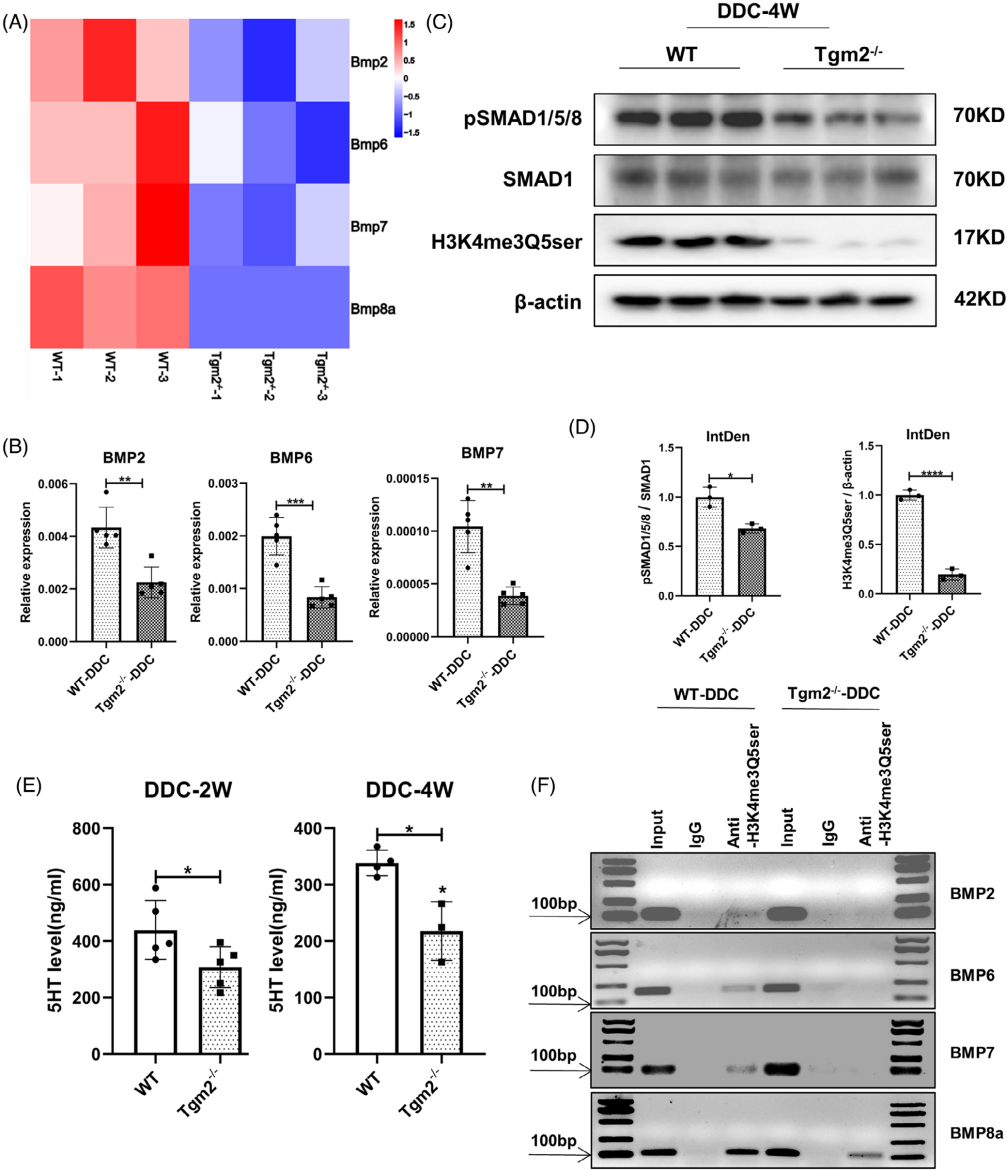
Tgm2 regulates H3K4me3Q5ser via serotonin, thus activating BMP signalling to regulate DR. (A) Heat map of BMP2, BMP6, BMP7, and BMP8a in WT and Tgm2^−/−^ mouse livers by RNA‐seq analysis (*n* = 3/group). (B) Hepatic expression levels of BMP2, BMP6, and BMP7 were determined by RT‐qPCR (*n* = 5/group). (C) Western blot assay of pSMAD1/5/8, SMAD1, H3K4me3Q5ser, and β‐actin in liver extracts from WT and Tgm2^−/−^ mice (*n* = 3/group). (D) Quantification of pSMAD1/5/8 and H3K4me3Q5ser WB signals measured as integrated density using ImageJ (*n* = 3/group). (E) Content of 5‐HT in Tgm2^−/−^ mouse after DDC‐2 W or DDC‐4 W injury (*n* = 3 ≥ group). (F) ChIP experiments on WT and Tgm2^−/−^ mouse liver tissues (*n* = 3/group). Comparisons between two groups were performed using two‐tailed Student's *t*‐test. *p < 0.05, **p < 0.01, ***p < 0.001, and ****p < 0.0001 represent four different levels of significant difference, respectively.

To elucidate the mechanism underlying Tgm2's regulation of DR, we performed a comparative analysis of gene expression profiles in the livers of WT and Tgm2‐deficient mice subjected to a 4‐week DDC‐induced cholestatic condition. RNA sequencing (RNA‐seq) revealed reduced expression of multiple BMP family genes in the Tgm2^−/−^ mouse liver following DDC‐induced injury (Figure 3A). The findings were further validated through RT‐qPCR and western blot assays (Figure 3B–D), indicating a significant suppression of BMP signalling in Tgm2^−/−^ mice. Then, we measured the expression of the BMPs in normal chow‐fed WT and Tgm2^−/−^ mice. The results showed that there was no significant difference in BMP2, BMP6, and BMP7 levels between WT and Tgm2^−/−^ mice fed with chow (Figure S4A). Additionally, the BMP signalling was neither activated nor inhibited in WT, Tgm2^−/−^, and DDC‐treated WT mice (Figure S4B,C). It suggests BMP‐Tgm2 axis may play a joint role in DDC‐induced cholestatic liver injury.

Next, we investigated the relationship between BMP signalling and Tgm2 in more detail. BECs can synthesize 5‐HT de novo and use 5‐HT as an autocrine/paracrine signalling to regulate biliary tree regeneration, thus participating in DR.^29^, ^30^ It has been reported that serotonylation in the nucleus can occur independently of 5‐HT receptors but is dependent on Tgm2. Tgm2 enzymatically adds 5‐HT to the glutamine residue at position 5 of histone H3. This results in the formation of a double‐modified histone, H3K4me3Q5ser, which involves the trimethylation of H3K4 and serotonylation of H3Q5 by Tgm2. The presence of K4me3 indicates an active transcriptional state of chromatin, which may be further enhanced by the double labelling of K4me/Q5ser.^31^, ^32^ Therefore, we hypothesized that Tgm2 regulated the deposition of H3K4me3Q5ser chromatin via 5‐HT, and this modified histone contributed to hepatocyte metaplasia through BMP signalling. To test our hypothesis, we examined the expression of H3K4me3Q5ser in the livers of WT and Tgm2^−/−^ mice. The results revealed a significant reduction in the expression of H3K4me3Q5ser in Tgm2^−/−^ mice (Figure 3C,D). Additionally, we measured the plasma 5‐HT levels and found that Tgm2^−/−^ mice exhibited lower levels of 5‐HT compared to WT mice following DDC‐induced injury (Figure 3E). Furthermore, chromatin immunoprecipitation (ChIP) experiments were conducted to confirm the interaction of H3K4me3Q5ser with BMP promoter elements. The results demonstrated that the anti‐H3K4me3Q5ser antibody precipitated DNA fragments containing BMP2, BMP6, BMP7, and BMP8a elements in the livers of WT mice, whereas precipitation of lesser or no DNA fragments was observed in Tgm2^−/−^ mouse liver (Figure 3F). Our results indicated that the knockout of Tgm2 reduced the binding of H3K4me3Q5ser to the promoters of BMP2, BMP6, BMP7, and BMP8a, thereby affecting their transcriptional activity. In summary, these results indicate that Tgm2 may activate BMP signalling by regulating H3K4me3Q5ser through serotonin.

### 2.4 Activation of BMP signalling promotes the development and maturation of BECs in DR to alleviate cholestasis in Tgm2^−/−^ mice

**FIGURE 4:**
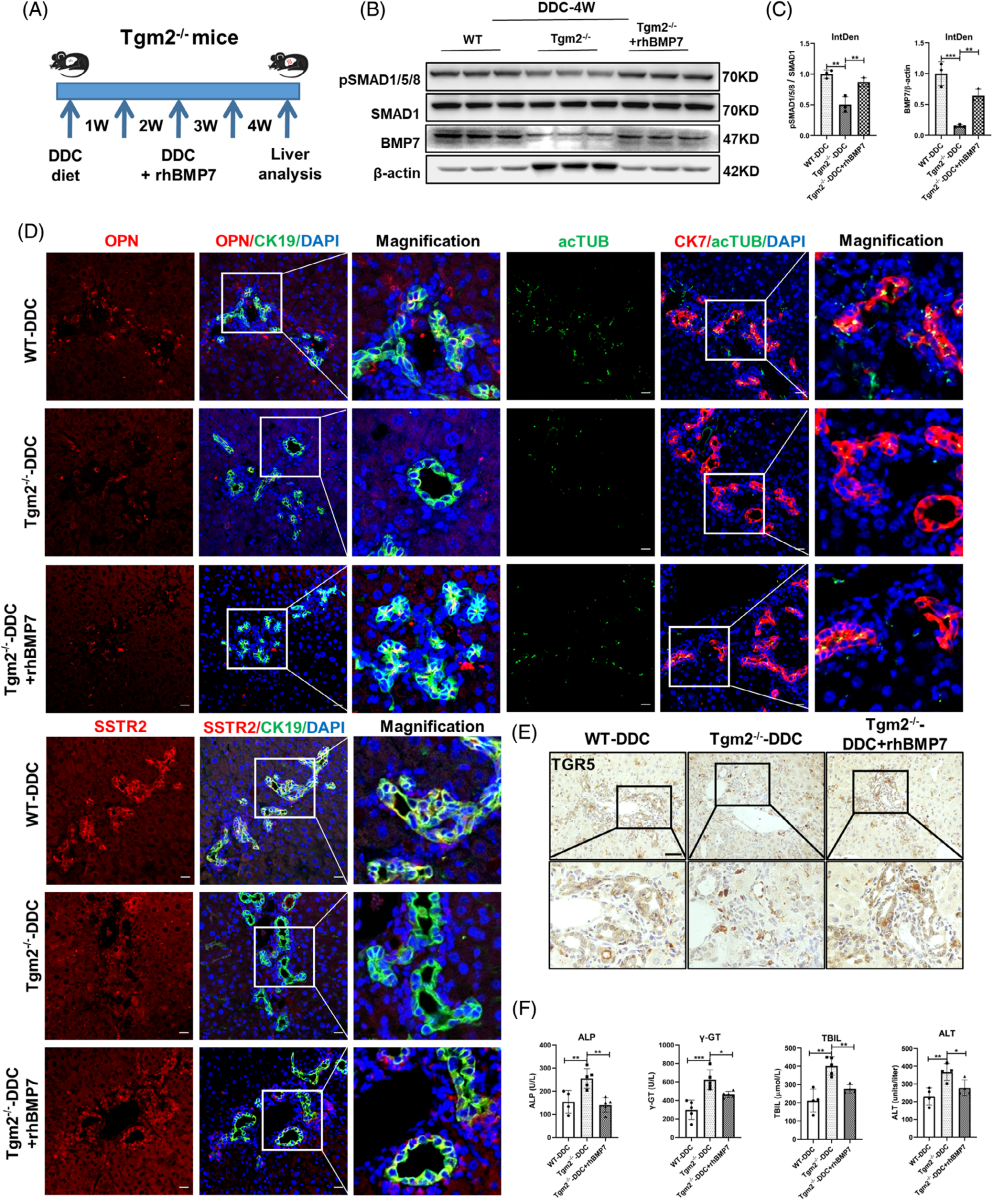
Activation of BMP signalling promotes the development and maturation of BECs to alleviate cholestasis in Tgm2^−/−^ mice. (A) Schematic diagram of the use of rhBMP7 in Tgm2^−/−^ mouse after 4‐week DDC injury. (B) Western blot assay of pSMAD1/5/8, SMAD1, BMP7, and β‐actin in liver extracts from WT and Tgm2^−/−^ mice (*n* = 3/group). (C) Quantification of pSMAD1/5/8 and BMP7 WB signals measured as integrated density using ImageJ (*n* = 3/group). (D) Co‐staining of CK19 and OPN or SSTR2 and co‐staining of CK7 and acTUB in mouse liver samples (*n* = 4/group). Scale bar, 20 μM. (E) Immunohistochemical staining of TGR5 was observed in WT, Tgm2^−/−^, and Tgm2^−/−^ + rhBMP7 groups after 4‐week DDC injury (*n* = 4/group). Scale bar, 50 μM. (F) Plasma ALP, γ‐GT, TBIL, and ALT levels were measured in WT and Tgm2^−/−^ mice (*n* ≥ 4/group). Comparisons between multiple groups were performed using ordinary one‐way ANOVA with Dunnett's multiple comparison test. Significant difference was presented at the levels of *p < 0.05, **p < 0.01, ***p < 0.001, and ****p < 0.0001.

In order to investigate whether BMP signalling is involved in the structural and functional development of BECs through Tgm2 in DR, we employed rhBMP7 recombinant proteins to activate BMP signalling. BMP signalling and FGF signalling, which coordinate and cooperate between two distinct mesodermal layers, play a crucial role in controlling liver development and specification.^33^ Besides, BMP signalling could regulate both BEC‐driven and hepatocyte‐driven liver regeneration.^34^, ^35^ In our study, rhBMP7 was used to activate BMP signalling in Tgm2^−/−^ mice during DDC‐4 W treatment (Figure 4A). The results demonstrated that BMP7 expression and phosphorylation of Smad1/5/8 were significantly decreased in Tgm2^−/−^ mice compared to WT mice. Moreover, the administration of rhBMP7 significantly enhanced BMP7 expression and promoted Smad1/5/8 phosphorylation (Figure 4B,C).

To assess the effects of BMP signalling activation on BECs, liver sections were immunostained for structural and functional markers specific to biliary cells, and the area of co‐staining markers was quantified following DDC‐induced injury. As shown in Figure 4D,E and Figure S5A,B, the expression of OPN, SSTR2, acTUB, and TGR5 in BECs of Tgm2‐deficient mice was significantly lower than that in WT mice. However, supplementation with rhBMP7 significantly reversed these reductions in marker expression. Additionally, the level of DDC‐induced cholestasis and hepatocyte injury was relieved in the Tgm2^−/−^ + rhBMP7 group, as indicated by reduced levels of ALP, γ‐GT, TBIL, and ALT (Figure 4F). Furthermore, Masson's staining results demonstrated that supplementation with rhBMP7 relieved the liver fibrosis caused by the absence of Tgm2 in DDC‐induced cholestasis (Figure S5C,D). The expression of desmin and αSMA also supported this conclusion (Figure S5E). These findings suggest that activation of BMP signalling has the potential to partially restore the integrity of proliferative BECs in DR and alleviate cholestatic liver injury induced by Tgm2 deficiency.

### 2.5 Inhibition of BMP signalling suppresses the metaplasia of hepatocytes, thereby affecting the development and maturation of BECs in DR

**FIGURE 5:**
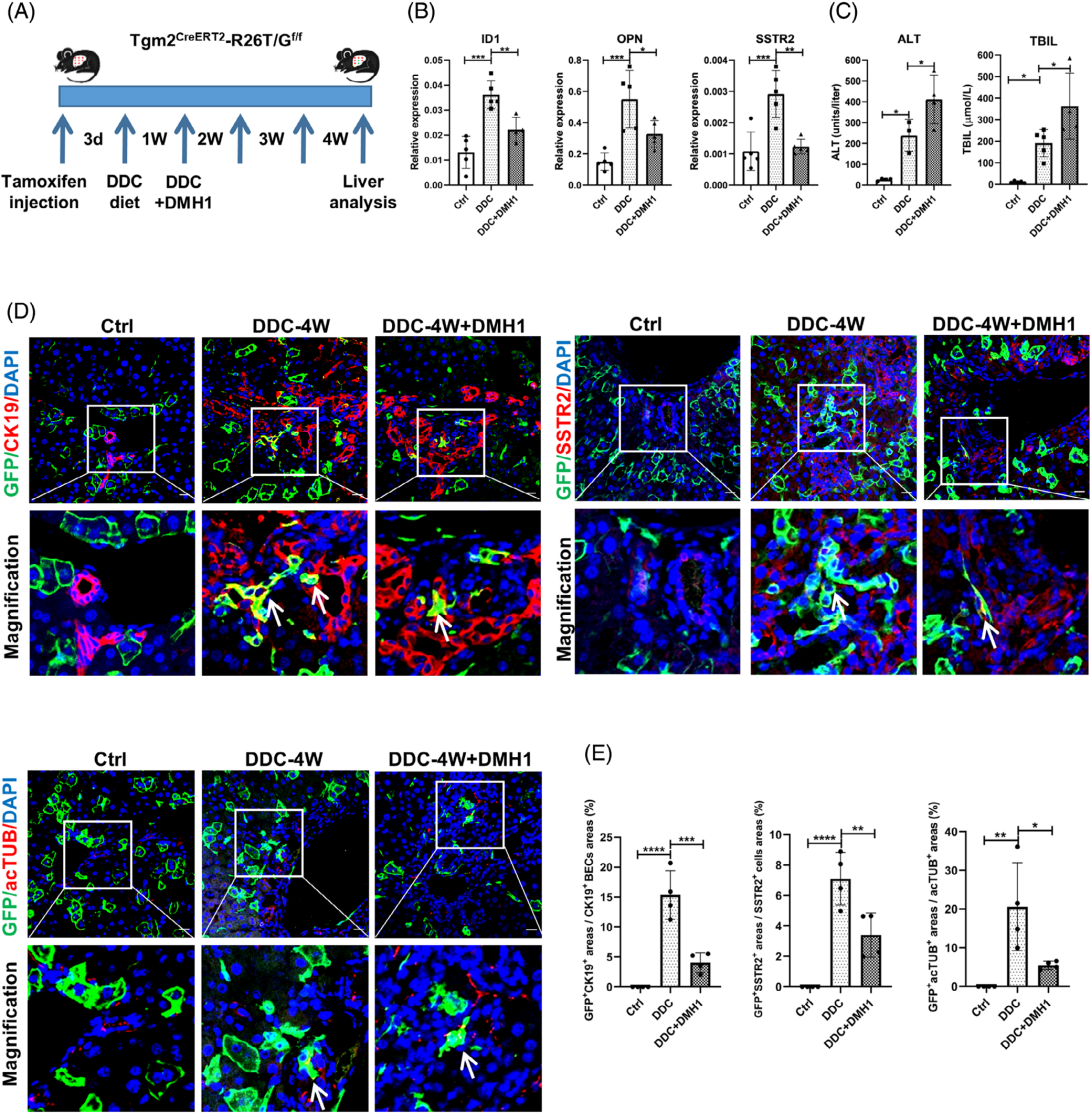
Inhibition of BMP signalling suppresses the metaplasia of hepatocytes, thereby affecting the development and maturation of BECs in DR. (A) Schematic diagram of hepatocyte fate tracing after 4‐week DDC diet and DMH1 injection in Tgm2^CreERT2^‐R26T/G^f/f^ mice. (B) Hepatic expression levels of ID1, OPN, and SSTR2 in Tgm2^CreERT2^‐R26T/G^f/f^ mouse liver (*n* ≥ 4/group). (C) ALT and TBIL levels were measured in Tgm2^CreERT2^‐R26T/G^f/f^ mice (*n* ≥ 3/group). (D) Immunofluorescence co‐staining of CK19, acTUB, and SSTR2 with the GFP lineage label was observed after 4‐week DDC treatment, but lesser co‐stained cells were observed after DMH1 injection (*n* = 4/group). White arrows indicated co‐stained cells. Scale bar, 20 μM. (E) The percentage of marker^+^ cells' (i.e., cells that stained positive for CK19 or SSTR2 or acTUB) fluorescence intensity was determined in GFP lineage label by fluorescence colocalization analysis (*n* = 4/group). Comparisons between multiple groups were performed using ordinary one‐way ANOVA with Dunnett's multiple comparison test. Significant difference was presented at the levels of *p < 0.05, **p < 0.01, ***p < 0.001, and ****p < 0.0001.

To further investigate the relationship between BMP signalling and Tgm2 in DR, dorsomorphin homologue 1 (DMH1) was utilized to suppress BMP signal. DMH1 is a specific small‐molecule inhibitor that blocks BMP signalling.^36^ In our study, we investigated the effect of DMH1 on hepatocellular metaplasia following DDC‐4 W diet treatment (Figure 5A). We observed that the introduction of DMH1 significantly blocked the expression of ID1, a canonical BMP target gene, indicating the inhibition of BMP signalling in Tgm2^CreERT2^‐R26T/G^f/f^ mice (Figure 5B). Interestingly, the inhibition of BMP signalling through DMH1 resulted in more severe liver injury, as evidenced by significantly higher levels of plasma ALT and TBIL (Figure 5C). Masson's staining and the expression of desmin and αSMA confirmed that the inhibition of BMP signalling led to more severe liver fibrosis (Figure S5F–H). Additionally, the expression of OPN and SSTR2, which are markers associated with BECs, was significantly reduced upon DMH1 treatment, indicating the downregulation of these markers (Figure 5B). Further analysis of marker co‐staining revealed that GFP^+^CK19^+^ cells, which represent Tgm2 hepatocyte‐derived BECs expressing acTUB and SSTR2, were detected. However, the presence of GFP^+^ hepatocyte‐derived BECs was significantly reduced after DMH1 treatment (Figure 5D,E). This indicates that DMH1 treatment impeded the metaplasia of GFP^+^ hepatocytes into BECs by blocking BMP signalling, resulting in more severe liver damage in Tgm2^CreERT2^‐R26T/G^f/f^ mice. Overall, our findings demonstrate that BMP signalling is necessary for Tgm2‐mediated hepatocyte metaplasia in DDC‐induced DR.

### 2.6 Inhibition of Tgm2 represses the transdifferentiation of hepatocytes, thus affecting the development and function of BECs in vitro

In vitro, we employed a collagen‐rich three‐dimensional (3D) matrix to simulate the conditions observed in chronic cholestatic liver injury, in which only hepatocytes without cholangiocytes were placed.^11^ This experimental setup has been utilized multiple times to elucidate the role of hepatocytes in DR.^37^ Transdifferentiation is a specific type of metaplasia that can be used in vitro to research the role of hepatocytes in DR.^12^ Mature hepatocytes can be transdifferentiated into BECs and be capable of regaining their original phenotypes following bile ductular transdifferentiation, which resembles the ‘metaplasia’ observed in vivo.^38^, ^39^

**FIGURE 6:**
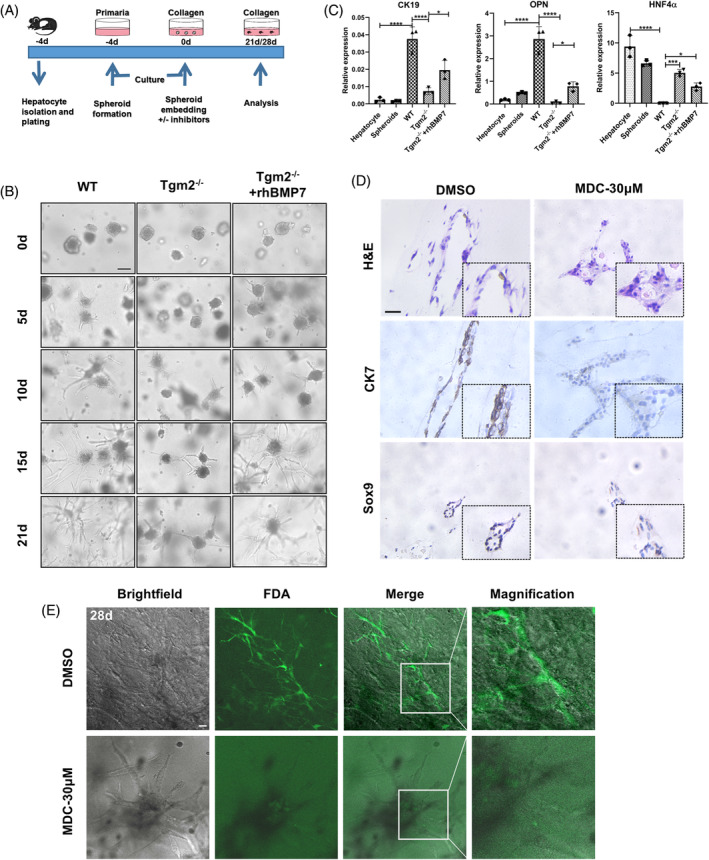
Inhibition of Tgm2 represses the transdifferentiation of hepatocytes, thus affecting the development and function of BECs in vitro. (A) Biliary transdifferentiation of adult hepatocytes in 3D culture. (B) Phase‐contrast micrographs of hepatocytic spheroids after being embedded in a collagen gel. The WT or Tgm2^−/−^ hepatocytes were cultured for 21 days in the absence or presence of rhBMP7 (200 ng/mL) (*n* = 3/group). Scale bar, 50 μM. (C) RT‐qPCR analysis was conducted to measure the mRNA expression of CK19, OPN, and HNF4α in the following groups: hepatocyte, spheroids, WT, Tgm2^−/−^, and Tgm2^−/−^ + rhBMP7 (*n* = 3/group). (D) H&E staining and immunohistochemical staining of CK7 and Sox9 showed ductular structures produced by hepatocytic spheroids after 21‐day collagen gel culture in the absence or presence of 30 μM MDC (*n* = 3/group). Scale bar, 20 μM. (E) Images of uptake and excretion of FDA by ductular structures after 28‐day hepatocytic spheroid collagen gel culture (*n* = 3/group). Scale bar, 20 μM. Comparisons between multiple groups were performed using ordinary one‐way ANOVA with Tukey's multiple comparison test. Significant difference was presented at the levels of *p < 0.05, **p < 0.01, ***p < 0.001, and ****p < 0.0001.

We first investigated whether the deletion of Tgm2 could affect the transdifferentiation of hepatocytes in 3D collagen gel matrix. Hepatocytes were isolated from WT and Tgm2^−/−^ mice (Figures 6A and S6A), and spherical aggregates of hepatocytes were embedded in a collagen gel for culturing, where they migrated into the gel and formed elongated structures. The Tgm2^−/−^ group mice exhibited reduced branching morphogenesis, characterized by fewer and simpler branches compared to the WT group, but the branching morphogenesis was enhanced by rhBMP7, with the appearance of more complex and finer branches in spherical aggregates of Tgm2^−/−^ hepatocytes (Figure 6B). These morphological changes are related to the expression of biliary and hepatocellular markers.

Hepatocytes isolated from WT mouse strongly expressed HNF4α, but they expressed minimal CK19 and OPN prior to culture. Compared to the WT group, the Tgm2^−/−^ group exhibited a significant decrease in the expression of CK19 and OPN, which were related to biliary differentiation, but hepatocyte marker HNF4α had a significant increase after 21 days of cultivation of spherical aggregates. However, the expression of CK19 and OPN was significantly increased in the Tgm2^−/−^ group in the presence of rhBMP7, while HNF4α was significantly reduced (Figure 6C). These results indicate that the absence of Tgm2 affected the transdifferentiation of hepatocyte in vitro and BMP7 partially rescued this process in Tgm2^−/−^ hepatocyte. Then, we used the Tgm2 inhibitor MDC to further study the role of the Tgm2 during the transdifferentiation of hepatocytes. MDC suppressed the tubular branching morphogenesis of hepatocytes cultured in 3D collagen gel matrix (Figure S6B). Additionally, H&E staining showed a decrease in the formation of tubular structures by hepatocyte spheroids with the addition of MDC. Subsequently, we examined the effect of MDC on the expression of various BEC markers through immunohistochemistry staining (Figures 6D and S6C). The results showed that the expression of CK7 and Sox9 increased in culture, but this increase was slowed down following MDC treatment. Previous studies have reported that the addition of fluorescein diacetate (FDA) dye rapidly accumulates in the lumens of biliary structures, indicating the formation of functional bile ducts.^40^ In the DMSO group, a significant amount of FDA was absorbed into the lumens within 10 min, while only minimal dye absorption occurred in the MDC‐treated group (Figure 6E and Movie S1). To further validate our findings, we employed another Tgm2 inhibitor, ZM39923 hydrochloride (ZM),^41^ and a BMP pathway inhibitor, DMH1. Both inhibitors suppressed the tubular branching morphogenesis of mouse hepatocytes cultured in a collagen gel matrix (Figure S6D). In summary, our results indicate that both Tgm2 signalling and BMP signalling are necessary for biliary differentiation and morphogenesis of hepatocytes.

## 3 DISCUSSION

In this study, we provide the first evidence that Tgm2 is a crucial factor in DR through governing BMP signalling and plays an indispensable role in cholestatic liver disease. Chronic cholestasis caused by impairment of biliary function can progress to hepatitis and cirrhosis even requiring liver transplantation.^42^ The expanded and remodelled BECs in DR perform as effective bile excretory conduits and constitute functional and complementary network of bile excretory ducts in damaged tissues where hepatic bile canaliculi were missing.^43^ According to reports, cell proliferation in DR provides excellent relief from cholestasis‐induced injury by acquiring a cholangiocyte‐like phenotype through hepatocellular metaplasia.^12^ We found that the deletion of Tgm2 affected the development and maturation of BECs in DDC‐induced DR. We focus on the biliary epithelial tissue in the DR, which has a more complex and dynamic structure; however, the molecular mechanisms underlying this process are still uncertain.

The intrahepatic biliary tree modifies and transports the canalicular bile via the secretion and reabsorption of BECs. Diseases that affect integrity and stability of the functional structure of these BECs underlie cholangiopathies.^44^ The excretive and absorptive functions of BECs are dramatically influenced a variety of channels and receptors expressed on the apical plasma membrane and primary cilia of BECs.^45^ Primary cilia marked acTUB and cholangiocyte apical polarity marked OPN are both involved in sustaining the function and homeostasis of BECs. Somatostatin interacting with SSTR2 regulates ductal bile secretion, and the deletion of SST2 inhibits the absorption of biliary fluid and disrupts bile formation.^46^ The bile acid receptor TGR5 is present on cholangiocytes, which facilitates bile secretion and maintains tight junction integrity, thus protecting cholangiocytes from bile acid toxicity.^47^ Though few significant differences in biliary development were found between Tgm2^−/−^ and WT mice in normal feeding, deficiency of Tgm2 in mice caused the damage of the hallmarks of normal biliary cells such as OPN, acTUB, TGR5, and SSTR2 and even more severe cholestasis in DDC‐induced DR. Thus, our results indicate that the deletion of Tgm2 has a significant impact on the structure and function of proliferated BECs in DR.

Tgm2 is a multifunctional enzyme with multiple biological functions and plays an important role in liver injury and repair.^15^, ^16^, ^17^, ^18^, ^19^ This study provides a novel Tgm2^CreERT2^‐R26T/G^f/f^ lineage‐tracing mouse model for tracking the fate of Tgm2^+^ cells, as shown that Tgm2 is mainly located in periportal hepatocytes. The sources of proliferative BECs in DR have been reported to be cholangiocytes, hepatic progenitor cells, or hepatocytes.^3^ Periportal hepatocytes can be involved in the normal homeostasis of ductular cells.^37^ Our results displayed the phenotypic plasticity of Tgm2^+^ mature hepatocytes. We constructed DDC‐4 W cholestatic liver injury model in Tgm2^CreERT2^‐R26T/G^f/f^ mice and found that Tgm2^+^ hepatocytes were converted into cholangiocytes. These reprogrammed cells displayed acTUB‐marked primary cilia, CK19‐marked mature biliary, and SSTR2‐marked functional biliary, indicating that hepatocytes can participate in DR by hepatocytic metaplasia in DDC‐induced cholestasis.

Several reports apply 3D collagen gel culture model to investigate the cellular origin of newly formed BECs in atypical DR.^37^ Mature hepatocytes can convert into BECs and are able to revert to their original phenotype after biliary transdifferentiation under certain conditions, similar to the ‘metaplasia’ observed in vivo.^38^, ^39^ In addition, our in vitro experimental results showed that within a collagen‐rich matrix, the knockout or inhibition of Tgm2 strongly suppressed the biliary differentiation of hepatocytes, especially by suppressing hepatocytic ductular morphogenesis. We also validated these findings by the rapid uptake and accumulation of FDA in the biliary structures, but only a little FDA absorbed and accumulated in Tgm2 blocking group, suggesting that Tgm2 was responsible for hepatocyte‐derived BECs in vitro.

Previous studies have revealed that various signalling pathways play an important role in DR including Notch, Hippo/YAP, and Wnt/β‐catenin.^3^ Considering that these signalling pathways have different effects on liver regeneration, more studies are needed to reveal the mechanisms of DR. BMPs belong to the TGFβ superfamily and are essential for hepatic specification.^33^ Besides, BMP signalling administrates BEC‐driven liver regeneration via Tbx2b and Id2a.^34^ We revealed that Tgm2 contributed to the DR and that the deletion of Tgm2 could significantly alter the reprogramming of the hepatocytes. Notably, our RNA‐seq results showed that many BMP family genes were downregulated in the Tgm2^−/−^ mice, while overexpression of the BMP7 protein rescued the phenotype of deficiency of biliary integrity and repaired hepatic cholestasis after Tgm2 was disrupted. Besides, DMH1 suppressed the BMP signalling, which facilitated the metaplasia of hepatocytes, thereby affecting the development and maturation of BECs and causing more severe liver damage in DR. Importantly, we revealed that Tgm2 regulated H3K4me3Q5ser by 5‐HT, thus activating BMP signalling to regulate DR. The deficiency of Tgm2 caused a significant decrease in 5‐HT content and H3K4me3Q5ser expression in DDC‐treated liver injury. 5‐HT is connected with the pathogenesis of numerous liver disorders, such as steatohepatitis, liver fibrosis, and cholangiocarcinoma, and acts as an autocrine/paracrine signalling to regulate the reconstruction of biliary tree.^29^, ^30^ Serotonin in the nucleus can be independent of the serotonin receptor but depends on Tgm2, which is essential for the formation of H3K4me3Q5ser. Interestingly, H3K4me3Q5ser is ubiquitous in mammalian tissues and enriched in the euchromatin territory. The function of H3K4me3Q5ser deposition is to promote and increase the expression of the genes in a variety of tissues and cells, thus facilitating cellular differentiation.^31^, ^32^, ^48^ We performed ChIP assays and confirmed the interaction between H3K4me3Q5ser and the BMP promoter elements. These results suggest that the Tgm2‐BMP signalling axis is a novel pathway involved in DR.

In conclusion, our studies reveal that Tgm2‐BMP signalling axis plays a key role in the structure and function of proliferated BECs during DR. To our knowledge, this study makes the first attempt to reveal that Tgm2 regulates BMP signalling activation to mediate hepatocyte metaplasia so as to restore liver injury. Tgm2 that acts as drivers for specific hepatocytes to initiate DR is a reliable reprogramming factor, which provides an innovative direction for gene therapy and regenerative medicine.

## 4 METHODS

### 4.1 Animal studies

The strains and animals used in our experiments were purchased from Biocytogen (Beijing, China) or Cyagen (Beijing, China) and bred in‐house, including Tgm2‐^CreERT2^ mice, ROSA26‐mTomato/mGFP (Rosa26‐mTmG) reporter mice (Biocytogen, Beijing, China), and Tgm2^−/−^ mice (Cyagen, Beijing, China). C57BL/6N mice were purchased from the Huazhong Agricultural University Experimental Animal Center and used as wild‐type (WT) mice.

To generate Tgm2‐knockout mice, the exon 2–4 fragments of Tgm2 gene of mice were knocked out by Cyagen Biosciences Inc. (China). The knockout started at exon 2 (about 0.53% of the coding region). Exons 2~4 covered 26.34% of the coding region. The Tgm2^−/−^ mice are total body knockout. In lineage‐tracing experiments, the P2A‐i^CreERT2^ cassette was inserted behind the stop codon of exon 13 of Tgm2, and the knock‐in mice were prepared based on the CRISPR/Cas9 system by Biocytogen. Tgm2‐^CreERT2^ mice were crossed with Rosa26‐mTmG mice to obtain Tgm2^CreERT2^‐R26T/G^f/f^ heterozygous mice. Genomic DNA was extracted from the ear of 4‐week‐old mouse for genotyping of mice, and PCR was carried out using the primers in Table 3. All the mice were maintained in standard, specific pathogen‐free facility of the Laboratory Animal Research Center of Huazhong Agricultural University at 22–24°C with a 12‐h dark/light cycle at the density of 4–5 mice per cage. All mouse health status was monitored on daily basis. In this study, 2‐month‐old male mice were used unless specified otherwise. All the mice were kept on a standard chow diet purchased from the Huazhong Agricultural University Experimental Animal Center. All animal experiment procedures were approved by the animal ethics and welfare committee of Huazhong Agricultural University.

### 4.2 In vivo experiments

In all experiments, mice received humane care and were sex and age matched. In DDC‐induced injury experiments, mice received 0.1% DDC (3,5‐diethoxycarbonyl‐1,4‐dihydrocollidine; Sigma, 137,030) for the indicated duration. Tamoxifen (Sigma, T5648) was diluted with corn oil (Aladdin, C116023) to reach 1 mg/mL final concentration, and the diluted tamoxifen was injected into mice at the dose of 5 mg/kg body weight. Tgm2^CreERT2^‐R26T/G^f/f^ mice were intraperitoneally injected with a single dose of tamoxifen once before DDC treatment.

In lineage‐tracing experiments, Tgm2^CreERT2^‐R26T/G^f/f^ mice were orally gavaged with either phosphate‐buffered saline (PBS, Beyotime, ST448‐1 L) or MDC (MCE, HY‐D1027, 25 mg/kg body weight) every day, simultaneously accompanied by 2‐week DDC feeding.

In the inhibition experiment of BMP signalling, DMH1 (MCE, HY‐12273, 5 mg/kg body weight) was dissolved in carrier reagents (PEG 400, Tween 80, physiological saline) and administered via intraperitoneal injection every other day for 3 weeks in Tgm2^CreERT2^‐R26T/G^f/f^ mice. In rescue experiment, rhBMP7 (PeproTech, 120‐03P) was dissolved in PBS and added with 5% trehalose. Afterwards, Tgm2^−/−^ mice were injected with rhBMP7 (100 g/kg body weight) three times a week for 2 weeks, whereas the WT mice were injected with PBS (5% trehalose). All experiments were continued for 4 weeks of DDC feeding. Detailed information on the commercial reagents and chemicals can be found in Table S1.

### 4.3 Biochemical index detection

Blood was placed into a polyethylene tube containing heparin and centrifuged at 1000 × g for 10 min at 4°C. All samples were kept on ice until centrifugation. Following centrifugation, plasma was harvested and stored at −80°C until analysis. According to the manufacturer's protocol, plasma ALP (Nanjing Jiancheng, A059‐2), γ‐GT (Nanjing Jiancheng, C017‐2), TBIL (Nanjing Jiancheng, C019‐1‐1), ALT (Nanjing Jiancheng, C009‐2), and AST (Nanjing Jiancheng, C010‐2) were determined to evaluate cholestatic liver injury using a Multiskan MK3 microplate reader (Thermo Electron Corporation, USA). All commercial kits' detailed information can be found in Table S1.

### 4.4 Histopathology

H&E staining and Masson's staining were performed for histological studies by using haematoxylin and eosin (Beyotime, C0105S) and Masson's trichrome staining kit (Solarbio, G1340). Liver tissues were fixed in 4% paraformaldehyde for a period of 24–48 h, processed by routine histology procedures, embedded in paraffin, sectioned into 4 μM pieces, and mounted on the slide. Thereafter, the sections were stained with H&E dye or Masson's trichrome staining reagents following the standard steps to evaluate the severity of the histopathology. Examinations were performed, and photographs were captured with light microscopes (MSHOT, MF52‐N, and Olympus BX53). Part of photographs were panoramically scanned by 3DHISTECH (CaseViewer, Hungary).

### 4.5 Immunofluorescence and immunohistochemistry

Immunofluorescence and immunohistochemistry were performed on 4‐μM sections of formalin‐fixed paraffin‐embedded tissues. Briefly, tissue sections were deparaffinized, followed by antigen unmasking by microwave heating for 20 min at 98°C in 10 mM sodium citrate pH 6.0 or Tris‐EDTA buffer pH 9.0. Sections were washed for 15 min in PBS solution before blocking for 30 min in PBS containing 10% goat serum. Thin sections were stained with primary antibodies GFP (1:100 dilution, Santa Cruz, sc‐9996, or Proteintech, 50,430), CK19 (1:100 dilution, Servicebio, GB12197), OPN (1:50 dilution, Servicebio, GB11500), acTUB (1:200 dilution, Sigma, T6793), SSTR2 (1:300 dilution, ABclonal, A15101), TGR5 (1:100 dilution, ELK Biotechnology; ES4906), HNF4α (1:100 dilution, Abcam, ab41898), αSMA (1:100 dilution, Servicebio, GB111364), desmin (1:100 dilution, Servicebio, GB12081), Col1a1 (1:100 dilution, Servicebio, GB11022‐3), EpCAM (1:150 dilution, Servicebio, GB1127), CK7 (1:200 dilution, Abcam, ab181598), and Sox9 (1:100 dilution, Millipore, AB5535) and incubated at 4°C for 12–18 h. For double immunohistochemical staining, Alexa Fluor 555 (1:500 dilution, Invitrogen, A‐21428) and Alexa Fluor 488 (1:500 dilution, Invitrogen, A11001) were used as secondary antibodies and incubated at room temperature for 1 h. DAPI (Abcam, ab104139) counterstaining was employed to demonstrate nuclei. Images were acquired with a laser scanning confocal microscope (LSM800, Carl Zeiss Microscopy) and analysed by ZEN software (ZEN 2.3 lite, Carl Zeiss Microscopy) with fixed parameters. For immunohistochemistry staining, the corresponding secondary antibody was Biotin‐conjugated Affinipure Goat Anti‐Rabbit IgG (H + L) (Proteintech, SA00004‐2) for CK7 and Sox9 at room temperature for 50 min. Finally, all sections were imaged using a light microscope (MF52‐N, MSHOT, and Olympus BX53) at a magnification of 20. All antibodies used in our study are listed in Table S2.

### 4.6 RNA sequencing and RT‐qPCR

The total RNA of the liver was extracted using the RNAiso Plus (Takara, 9109) in WT and Tgm2^−/−^ mice after 4 weeks of DDC diet and subjected to Biomarker Technologies Corporation (Beijing, China) for RNA sequencing. All usable reads were uniquely mapped to a gene that was used to evaluate the expression level. The DEGseq R package was used to analyse differentially expressed genes in two samples based on the conditions of a fold change (FC) ≥ 1.5.

The RNAiso Plus (Takara, 9109) was used to isolate total RNA. Then, the first‐strand cDNA was synthesized using the PrimeScript RT Reagent Kit with gDNA Eraser (Takara, RR047A). Real‐time PCR was performed using the MonAmp™ SYBR® Green qPCR Mix (Monad, MQ10201S, Low ROX). Real‐time PCR was measured using QuantStudio™ 3 Real‐Time PCR Instrument (Applied Biosystems, USA). The relative levels were calculated using the comparative Ct method (2^−ΔΔCt^ method). All primer sequences are listed in Table S3.

### 4.7 Western blots

For whole‐cell protein extraction, liver tissues were prepared in lysis buffer (Beyotime, P0013B). Protein lysates were separated by SDS‐PAGE. Next, the gel was transferred to polyvinylidene difluoride membranes (Millipore, IPVH00010). After being blocked with 5% skimmed milk, the membranes were incubated overnight with the anti‐p‐SMAD1/5/8 (1:1000 dilution, ABclonal, AP0850), anti‐p‐SMAD1 (1:1000 dilution, Abcam, ab126761), anti‐H3K4me3Q5ser (1:1000 dilution, Sigma, ABE2580), anti‐BMP7 (1:1000 dilution, ABclonal, A0697), anti‐β‐actin (1:2000 dilution, Santa Cruz, sc‐58,673), anti‐Tgm2 (1:1000 dilution, Abcam, ab2386), and anti‐GAPDH (1:5000 dilution, Proteintech, 60,004‐1‐Ig) at 4°C for 12 h. Then, the membranes were incubated with the corresponding secondary antibodies (Biotin‐conjugated Affinipure Goat Anti‐Rabbit IgG (H + L), 1:5000 dilution, Proteintech, SA00004‐2; Biotin‐conjugated Affinipure Goat Anti‐Mouse IgG (H + L), 1:5000 dilution, Proteintech, SA00004‐1) at room temperature for 1.5 h. Finally, the membranes were visualized with enhanced chemiluminescence (Beyotime, P0018S).

### 4.8 ChIP assay

To perform ChIP assay, the liver tissues of WT and Tgm2^−/−^ mice were weighed and transferred into 2‐mL tube. Then, the samples were homogenized at a frequency of 45 Hz for 15 s at 4°C using tissue grinder (Scientz‐48) in cold PBS containing 1 mM PMSF. Following centrifugation at 8000 rpm for 2 min at 4°C, the cell pellets were resuspended in PBS containing formaldehyde (1% final) and DNA‐protein crosslinking was allowed to occur at room temperature for 15 min with rotation. Crosslinking reaction was quenched by the addition of glycine to 125 mM. Samples were then centrifuged at 8000 rpm for 2 min at 4°C, and the cell pellets were washed twice with cold PBS containing 1 mM PMSF and resuspended in SDS lysis buffer (50 mM Tris–HCl, pH 8.0, 10 mM EDTA, 1% SDS) supplemented with PMSF and protease inhibitors for 10 min. Thereafter, samples were sonicated to shear chromatin into 500–800 bp fragments utilizing ultrasonicator (Covaris, M220 Focused‐ultrasonicator), and the sonication parameters are as follows: peak incident power: 75w; duty factor: 10%; cycles per burst: 200; and duration: 900 s. Sonication products were centrifuged at 13000 rpm for 5 min at 4°C. For each sample, 200 μL supernatant was transferred into a new 2‐mL tube and diluted to 2 mL with ChIP dilution buffer (1.1% Triton X‐100, 1.2 mM EDTA, 167 mM NaCl, 16.7 mM Tris–HCl, pH 8.0). 35 μL Protein A/G Magnetic Beads (MCE, HY‐K0202‐1) were exploited in preclear process for 1 h 30 min at 4°C with rotation, and the supernatant was collected through magnetic separation. Subsequently, 200 μL of each precleared sample was obtained as 10% input, and the remaining sample was divided into two equal portions for overnight immunoprecipitation with Anti‐Histone H3Kme3Q5Ser Polyclonal Antibody (Millipore, ABE2580‐100UL) as target enriched material and rabbit IgG (Beyotime, A7016) as negative control with rotation. Antibody–chromatin complexes were precipitated with 30 μL Protein A/G Magnetic Beads (MCE, HY‐K0202) at 4°C for 2 h with rotation. Supernatant was then discarded, and collected beads were washed sequentially for 5 min at 4°C with three different buffers requiring magnetic separation at 4°C after each wash. Initially, low‐salt wash buffer (1% Triton X‐100, 2 mM EDTA, pH 8.0, 20 mM Tris–HCl, pH 8.0), then high‐salt wash buffer (1% Triton X‐100, 0.1% SDS, 500 mM NaCl, 2 mM EDTA, pH 8.0, 20 mM Tris–HCl, pH 8.0), and finally LiCl immune complex wash buffer (1% NP‐40, 0.25 mM LiCl, 1% Na‐deoxycholate, 1 mM EDTA, pH 8.0, 10 mM Tris–HCl, pH 8.0) were used. The beads were next washed twice with TE buffer (10 mM Tris–HCl, pH 7.5, 1 mM EDTA, pH 8.0). Antibody–chromatin complexes were eluted using the elution buffer (1% SDS, 100 mM NaHCO3). Consequently, all samples (including 10% inputs) were incubated at 65°C overnight for decrosslinking and DNA was extracted using Cycle Pure Kit (Omega, D6492). The ChIP‐isolated DNA was subjected to PCR amplification using the primer pairs.

### 4.9 Isolation and culture of hepatocytes

The mice were anaesthetized with Avertin (Tribromoethanol, Sigma, T48402, 240 mg/kg body weight) by intraperitoneal injection. The liver perfusion was done by injecting needle into the portal vein and providing the following solutions sequentially: 50 mL of EBSS (Sigma, E6276) supplemented with 0.5 mM EGTA (Sigma, E3889) and then 50 mL of HBSS (Sigma, H1641) supplemented with 100 U/mL of collagenase IV (Invitrogen, 17,101–015) and 0.05 mg/mL of trypsin inhibitor (Sigma, T2011). The perfused liver was carefully taken out, put onto a petri dish, added 25 mL of hepatocyte wash media (Invitrogen, 17,704–024), and massaged with two cell scrapers until the liver has become apart with only connective tissue left behind. Dissociated cells were passed through funnel with mesh into 50 mL of centrifugal tube. After centrifugation at 900 rpm for 5 min, cell pellet was resuspended in hepatocyte wash media, which were carefully overlaid with Percoll (Sigma, P4937) solution (90%). After centrifugation at 900 rpm for 10 min, harvested cell pellet was washed twice with Williams' E medium (Gibco, A1217601) and then suspended in Williams' E medium supplemented with 1% penicillin/streptomycin, 10 mmol/L nicotinamide (MCE, HY‐B0150), 10 ng/mL epidermal growth factor (EGF, PeproTech, 315–09), and 10^−7^ mol/L insulin (Sigma, I2643).

We performed a 3D collagen gel culture of mouse hepatocytes. We plated isolated hepatocytes onto Primaria dishes (Corning, 3471) to form spheroidal aggregates. After 4 days, we harvested spheroidal aggregates by gentle pipetting and centrifuged at a low speed. Then, we washed the pellet one time with 10% FBS Williams' E medium (Gibco, A1217601) and kept the tube containing spheroids on ice. Next, we mixed eight parts of Cellmatrix type I‐A (Nitta Gelatin, 637–00653), one part of concentrated 10× Williams' E medium (including the powder of Williams' E medium and 1.22 g of nicotinamide in 100 mL of distilled and deionized water. Caisson, WMP06), one part of the reconstruction buffer (2.2 g NaHCO_3_ in 100 mL of 0.05 N NaOH and 200 mM HEPES), and two parts of Williams' E medium (Gibco, A1217601). All the ingredients were ice‐cold, and the neutralized collagen mixture was kept on ice so as to avoid premature gelling. Finally, the neutralized collagen mixture was put into the tube containing spheroids and the spheroids were resuspended by gentle pipetting. Then, 200 μL resuspended spheroids were added to the centre of the 48‐well plastic plates in a CO_2_ incubator at 37°C for 10 min for gelling.

After the cells were embedded within the collagen gels, spheroidal aggregates were cultured in William's medium E supplemented with 10% FBS (Gibco, 12,484,028), 10 mmol/L nicotinamide (MCE, HY‐B0150), 10 ng/mL epidermal growth factor (EGF, PeproTech, 315–09), 10^−7^ mol/L insulin (Sigma, I2643), and 10 ng/mL tumour necrosis factor‐α (TNF‐α, PeproTech, 315‐01A), as well as various indicated inhibitors. In some experiments, the specific Tgm2 inhibitors, MDC (MCE, HY‐D1027, 30 or 50 μM) or rhBMP7 (PeproTech, 120‐03P, 200 ng/mL) or ZM39923 hydrochloride (MCE, HY‐12589, 50 or 100 μM), or a BMP signalling inhibitor DMH1 (MCE, HY‐12273, 100 μM) was added to the medium. The cell culture medium was changed every 2 days. After completion of culture, the gels were washed three times with PBS and then were either snap‐frozen for biochemical analyses or fixed in 4% paraformaldehyde, embedded in paraffin, sectioned, and stained with haematoxylin and eosin (Beyotime, C0105S) or subjected to immunocytochemistry.

Fluorescein diacetate (FDA, HY‐D0719) bought from MCE was added to the culture medium to delineate lumen structures formed by cultured hepatocytes. In our preliminary studies, cells were treated with 10 μM FDA and this dye was secreted into the lumens within 10 min in DMSO‐treated group, but only a little dye got into the lumens in MDC‐treated group. Photographs were captured with a light microscope (MF52‐N, MSHOT, China), and images of differentiated spheroidal aggregates treated with FDA were acquired with a laser scanning confocal microscope (LSM800, Carl Zeiss Microscopy) and analysed by ZEN software (ZEN 2.3 lite, Carl Zeiss Microscopy).

### 4.10 Quantitative and statistical analysis

Statistical analyses were performed using the GraphPad Prism (GraphPad 8.0). We entered replicate values and stacked them into columns. Each bar chart displayed data points. Comparisons between two groups were performed using two‐tailed Student's *t*‐test. Comparisons between multiple groups were performed using ordinary one‐way ANOVA with Dunnett's or Tukey's multiple comparison test. Statistical significance was presented at the level of *p < 0.05, **p < 0.01, ***p < 0.001, and ****p < 0.0001. All experiments were independently repeated using at least three mice per experimental group. The exact numbers of data and images used for each of the experiments are indicated in legends. Immunostained liver sections were quantified using ImageJ for the quantification of positive area expression.

## AUTHOR CONTRIBUTIONS

Lisheng Zhang and Yaqing Chen designed and analysed the study. Yaqing Chen and Yi Yan performed most of the experiments. Yaqing Chen performed primary hepatocyte isolation and cell experiments. Yujing Li, Liang Zhang, Tingting Luo, and Xinlong Zhu carried out part of the cell and animal experiments. Dan Qin and Ning Chen provided assistance in molecular assays. Wendong Huang, Xiangmei Chen, Liqiang Wang, and Xianmin Zhu revised the manuscript critically for important intellectual content. Lisheng Zhang and Yaqing Chen contributed to project planning and manuscript writing. All authors have read and provided comments on the manuscript.

## FUNDING INFORMATION

The project is supported by National Natural Science Foundation of China 32071143, National Key R&D Plan No. 2017YFA0103200 and 2017YFA0103202.

## CONFLICT OF INTEREST STATEMENT

The authors declare no conflict of interest.

## Supporting information


**Figure S1.** Tgm2^−/−^ mice develop normally without discernible defects or damage in the liver. (A) Generation of Tgm2^−/−^ mice by knocking out the exons 2–4 in the genome region. (B) Western blot assay of Tgm2 and GAPDH in liver extracts from WT and Tgm2^−/−^ mice after DDC‐4 W injury (*n* = 4/group). (C) Genotyping by PCR screening of genomic DNA using primers (*n* = 4/group). (D) Hepatic expression level of Tgm2 in WT and Tgm2^−/−^ mice after DDC‐4 W injury (*n* = 4/group). (E) Plasma TBIL was measured in chow‐fed WT and Tgm2^−/−^ mice (*n* = 3/group). (F) H&E staining in chow‐fed WT and Tgm2^−/−^ mice (n = 3/group). Scale bar, 200 μM. (G) Immunofluorescence co‐staining of OPN and CK19, EpCAM and CK19, acTUB and CK7, and OPN and HNF4α in chow‐fed WT and Tgm2^−/−^ mice (*n* = 3/group). Scale bar, 20 μM. Comparisons between two groups were performed using two‐tailed Student's *t*‐test. *p < 0.05, **p < 0.01, ***p < 0.001, and ****p < 0.0001 represent four different levels of significant difference, respectively.

**Figure S2.** Deletion of Tgm2 affects the authenticity and maturity of cholangiocytes in DDC‐induced DR (A) The percentage of marker^+^ cells' (i.e., cells that stained positive for OPN, SSTR2, or acTUB) fluorescence intensity was determined in CK19^+^ or CK7^+^ BECs by fluorescence colocalization analysis after 4‐week DDC injury (*n* = 4/group). (B) Quantification of the percentage of TGR5^+^ area in immunohistochemical staining (*n* = 4/group). (C) Co‐staining of the hepatic progenitor markers SOX9 and EpCAM with CK19 in 4‐week DDC‐induced mouse livers (*n* = 4/group). Scale bar, 20 μM. (D) Quantification of the percentage of CK19 + SOX9+ cells and CK19 + EpCAM+ areas in immunofluorescence staining (*n* = 4/group). (E) Schematic diagram of WT and Tgm2^−/−^ mice after 2‐week DDC liver injury. (F) Plasma ALT was measured in WT and Tgm2^−/−^ mice after 2‐week DDC injury (*n* = 4/group). (G) Hepatic expression levels of the OPN and SSTR2 were determined in WT and Tgm2^−/−^ mice after 2‐week DDC injury (*n* = 5/group). (H) Co‐staining of OPN and SSTR2 with the cholangiocyte marker CK19 and co‐staining of acTUB with the cholangiocyte marker CK7 were observed after 2‐week DDC injury (*n* = 3/group). Scale bar, 20 μM. Comparisons between two groups were performed using two‐tailed Student's *t*‐test. *p < 0.05, **p < 0.01, ***p < 0.001, and ****p < 0.0001 represent four different levels of significant difference, respectively.

**Figure S3.** MDC treatment suppresses the metaplasia of hepatocytes in DR (A) Schematic diagram of generation of Tgm2‐^CreERT2^ mice and the progeny of Tgm2^CreERT2^‐R26T/G^f/f^ mice. (B) Schematic diagram of hepatocyte fate tracing during 4‐week DDC injury in Tgm2^CreERT2^‐R26T/Gf/f mice. (C) Co‐staining of the CK19 and HNF4α with the GFP lineage label in the liver of normal or DDC‐fed mice after the injection of corn oil (*n* = 3/group). Scale bar, 10 μM. (D) Co‐staining of the hepatic stellate cell markers desmin, Col1a1, and αSMA with the GFP lineage label in normal adult mouse liver (*n* = 3/group). Scale bar, 20 μM. (E) Hepatic Tgm2 was measured in Tgm2^CreERT2^‐R26T/G^f/f^ mice after DDC injury by western blot (*n* = 3/group). (F) Masson's staining of liver samples after 4‐week DDC injury (*n* = 3/group). Scale bar, 100 μM. (G) Quantification of the percentage of collagen‐positive staining areas in Masson's staining (*n* = 4/group). (H) Hepatic expression levels of OPN and EpCAM were determined in Tgm2^CreERT2^‐R26T/G^f/f^ mice by RT‐qPCR analysis (*n* = 5/group). Comparisons between multiple groups were performed using ordinary one‐way ANOVA with Dunnett's multiple comparison test. Significant difference was presented at the levels of *p < 0.05, **p < 0.01, ***p < 0.001, and ****p < 0.0001.

**Figure S4.** There was no significant difference in BMP signalling between chow‐fed WT and Tgm2^−/−^ mice (A) Hepatic expression levels of BMP2, BMP6, and BMP7 were determined by RT‐qPCR (*n* ≥ 4/group). (B) Western blot assay of pSMAD1/5/8, SMAD1, and β‐actin in liver extracts from WT and Tgm2^−/−^ mice (*n* = 3/group). (C) Quantification of pSMAD1/5/8 measured by integrated density using ImageJ (*n* = 3/group). Comparisons between multiple groups were performed using ordinary one‐way ANOVA with Tukey's multiple comparison test. Comparisons between three groups were performed using ordinary one‐way ANOVA with Dunnett's multiple comparison test. Significant difference was presented at the levels of *p < 0.05, **p < 0.01, ***p < 0.001, and ****p < 0.0001.

**Figure S5.** Tgm2 signalling and BMP signalling influence the degree of liver fibrosis in DDC‐induced DR (A) The percentage of marker^+^ cells' (i.e., cells that stained positive for OPN, SSTR2, or acTUB) fluorescence intensity was determined in CK19^+^ or CK7^+^ BECs by fluorescence colocalization analysis after 4‐week DDC injury (*n* = 4/group). (B) Quantification of the percentage of TGR5^+^ area in immunohistochemical staining (*n* = 4/group). (C) Masson's staining in WT, Tgm2^−/−^, and Tgm2^−/−^ + rhBMP7 groups (*n* = 4/group). Scale bar, 50 μM. (D) Quantification of the percentage of collagen‐positive staining areas in Masson's staining (*n* = 4/group). (E) Hepatic expression levels of desmin and αSMA were determined in WT, Tgm2^−/−^, and Tgm2^−/−^ + rhBMP7 groups after 4‐week DDC injury (*n* = 4/group). (F) Masson's staining in Ctrl, DDC, and DDC + DMH1 groups (*n* = 4/group). Scale bar, 50 μM. (G) Quantification of the percentage of collagen‐positive staining areas in Masson's staining (*n* = 4/group). (H) Hepatic expression levels of desmin and αSMA were determined in Ctrl, DDC, and DDC + DMH1 groups after 4‐week DDC injury (*n* = 4/group). Comparisons between multiple groups were performed using ordinary one‐way ANOVA with Dunnett's multiple comparison test. Significant difference was presented at the levels of *p < 0.05, **p < 0.01, ***p < 0.001, and ****p < 0.0001.

**Figure S6.** Suppression of Tgm2 signalling or BMP signalling represses the transdifferentiation of hepatocytes, thus affecting the development and function of BECs in vitro (A) Phase‐contrast micrographs of freshly isolated hepatocytes. Scale bar, 50 μM. (B) Phase‐contrast micrographs of hepatocytic spheroids after being embedded in a collagen gel. The hepatocyte collagen gel culture was performed for 21 days in the absence or presence of MDC (30 or 50 μM) (*n* = 3/group). Scale bar, 50 μM. (C) The percentage of CK7^+^ or SOX9^+^ cells in total cells were measured in 3D collagen gel sections (*n* = 4/group). (D) Phase‐contrast micrographs of hepatocytic spheroids after 21‐day culture in a collagen gel in the absence or presence of ZM (50/100 μM) or DMH1 (100 μM) (*n* = 3/group). Comparisons between two groups were performed using two‐tailed Student's *t*‐test. *p < 0.05, **p < 0.01, ***p < 0.001, and ****p < 0.0001 represent 4 different levels of significant difference, respectively.

**Table S1.** Reagents and chemicals used for this study.
**Table S2.** Antibodies used for this study.
**Table S3.** List of primers used for genotyping and RT‐qPCR.

**Movie S1.** Video of 3D reconstruction of hepatocyte‐to‐biliary transdifferentiation in a collagen gel culture in vitro.

## Data Availability

The data that support the findings of this study are available from the corresponding author upon reasonable request.
