# Supplementary figures and images for: Deletion of Tgm2 suppresses BMP‐mediated hepatocyte‐to‐cholangiocyte metaplasia in ductular reaction
Source: Cell Prolif. 2024 Apr 16;57(10):e13646. doi: 10.1111/cpr.13646 (PMC11471396; doi:10.1111/cpr.13646)

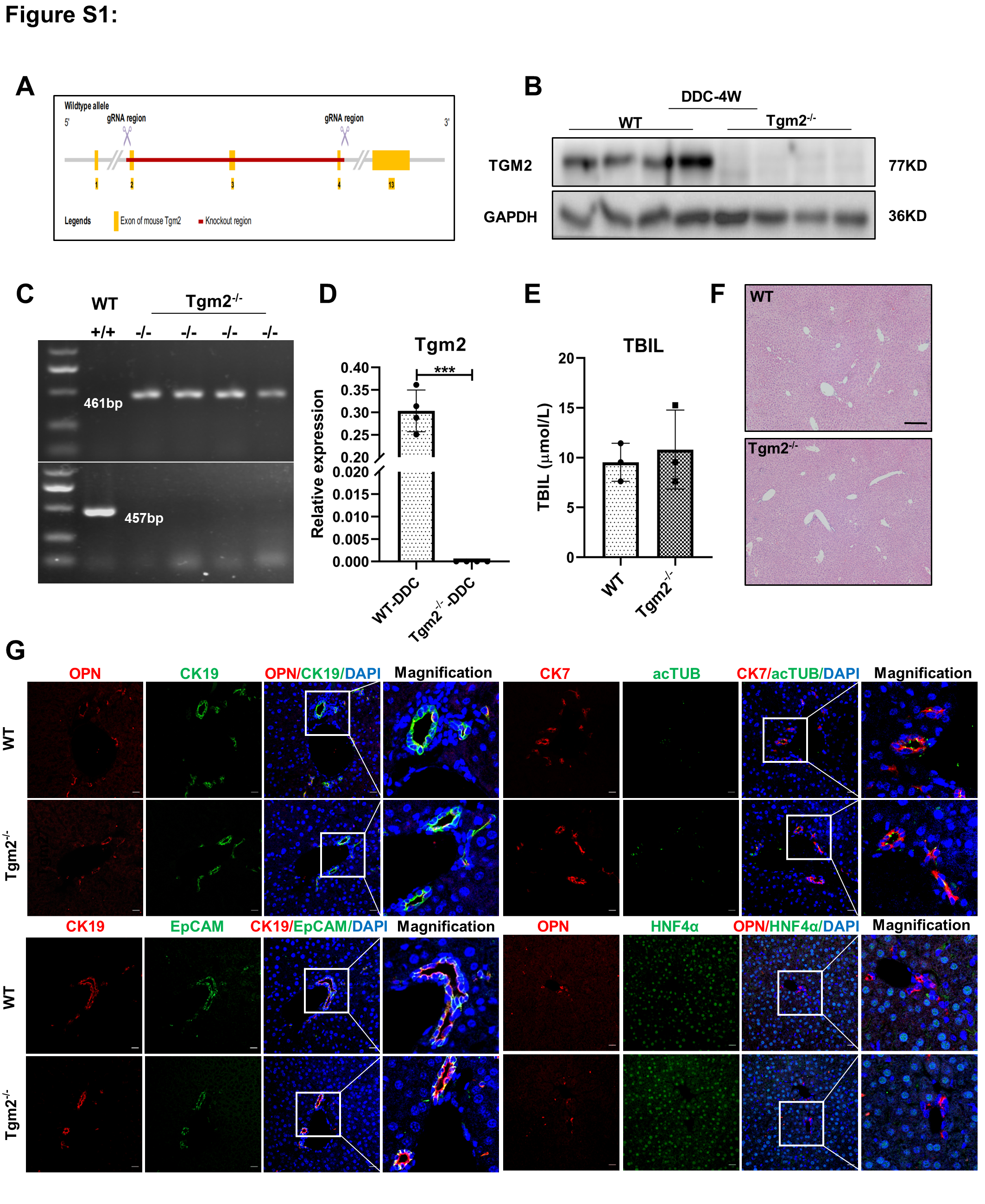

Supplement: Supplementary file 1 — Figure S1. Tgm2−/− mice develop normally without discernible defects or damage in the liver. (A) Generation of Tgm2−/− mice by knocking out the exons 2–4 in the genome region. (B) Western blot assay of Tgm2 and GAPDH in liver extracts from WT and Tgm2−/− mice after DDC‐4 W injury (n = 4/group). (C) Genotyping by PCR screening of genomic DNA using primers (n = 4/group). (D) Hepatic expression level of Tgm2 in WT and Tgm2−/− mice after DDC‐4 W injury (n = 4/group). (E) Plasma TBIL was measured in chow‐fed WT and Tgm2−/− mice (n = 3/group). (F) H&E staining in chow‐fed WT and Tgm2−/− mice (n = 3/group). Scale bar, 200 μM. (G) Immunofluorescence co‐staining of OPN and CK19, EpCAM and CK19, acTUB and CK7, and OPN and HNF4α in chow‐fed WT and Tgm2−/− mice (n = 3/group). Scale bar, 20 μM. Comparisons between two groups were performed using two‐tailed Student's t‐test. *p < 0.05, **p < 0.01, ***p < 0.001, and ****p < 0.0001 represent four different levels of significant difference, respectively. [file CPR-57-e13646-s006.tif]

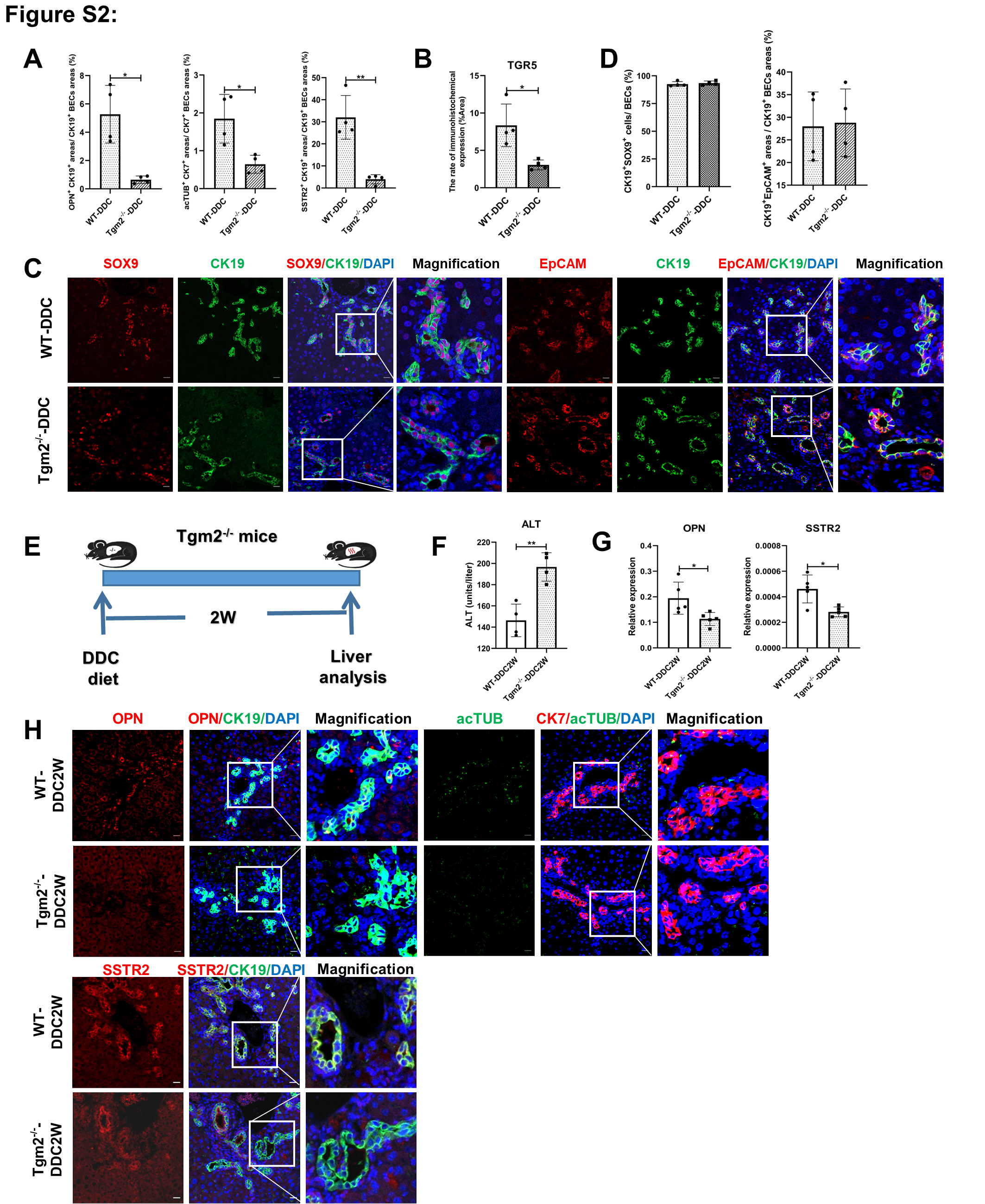

Supplement: Supplementary file 2 — Figure S2. Deletion of Tgm2 affects the authenticity and maturity of cholangiocytes in DDC‐induced DR (A) The percentage of marker+ cells' (i.e., cells that stained positive for OPN, SSTR2, or acTUB) fluorescence intensity was determined in CK19+ or CK7+ BECs by fluorescence colocalization analysis after 4‐week DDC injury (n = 4/group). (B) Quantification of the percentage of TGR5+ area in immunohistochemical staining (n = 4/group). (C) Co‐staining of the hepatic progenitor markers SOX9 and EpCAM with CK19 in 4‐week DDC‐induced mouse livers (n = 4/group). Scale bar, 20 μM. (D) Quantification of the percentage of CK19 + SOX9+ cells and CK19 + EpCAM+ areas in immunofluorescence staining (n = 4/group). (E) Schematic diagram of WT and Tgm2−/− mice after 2‐week DDC liver injury. (F) Plasma ALT was measured in WT and Tgm2−/− mice after 2‐week DDC injury (n = 4/group). (G) Hepatic expression levels of the OPN and SSTR2 were determined in WT and Tgm2−/− mice after 2‐week DDC injury (n = 5/group). (H) Co‐staining of OPN and SSTR2 with the cholangiocyte marker CK19 and co‐staining of acTUB with the cholangiocyte marker CK7 were observed after 2‐week DDC injury (n = 3/group). Scale bar, 20 μM. Comparisons between two groups were performed using two‐tailed Student's t‐test. *p < 0.05, **p < 0.01, ***p < 0.001, and ****p < 0.0001 represent four different levels of significant difference, respectively. [file CPR-57-e13646-s005.tif]

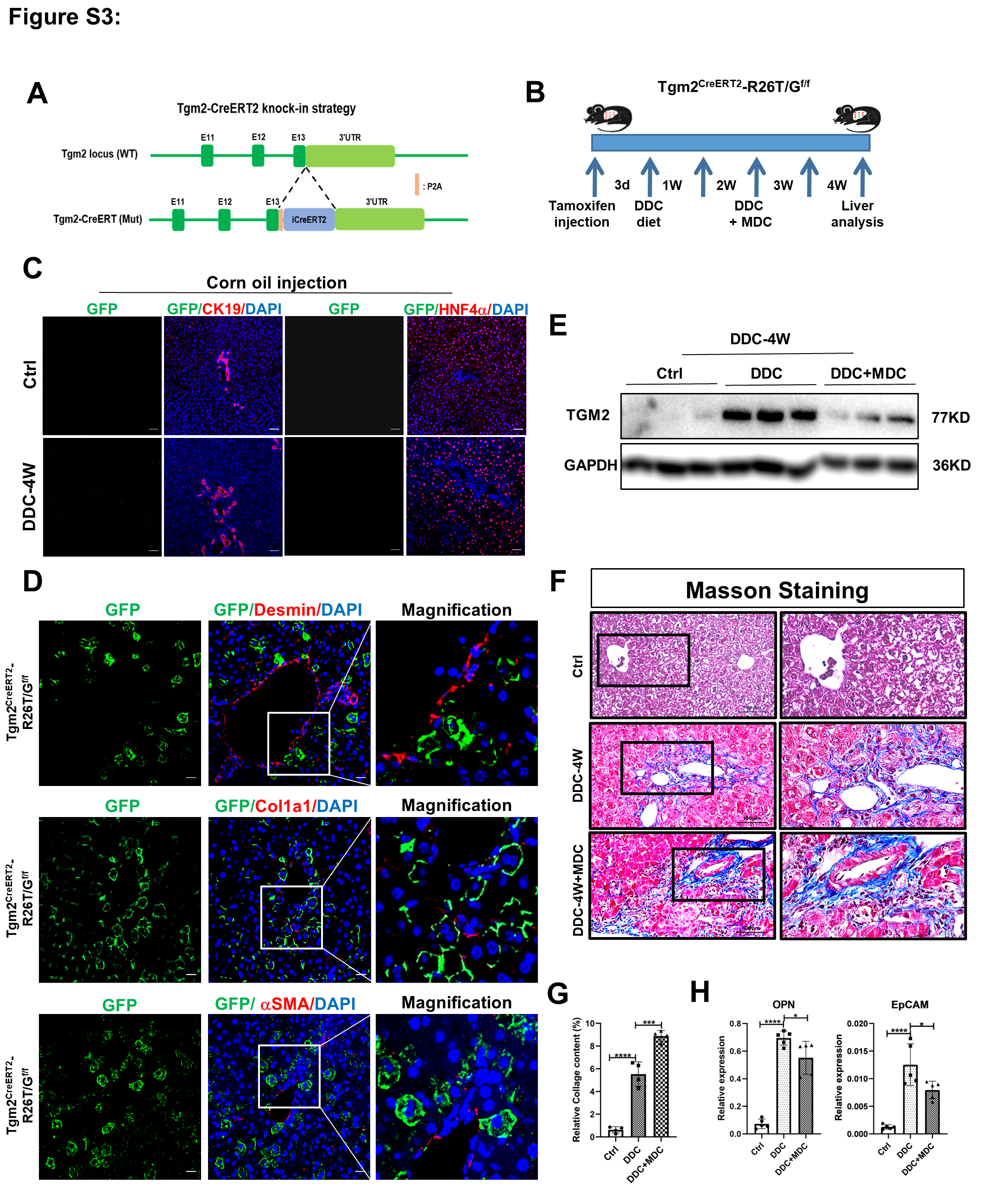

Supplement: Supplementary file 3 — Figure S3. MDC treatment suppresses the metaplasia of hepatocytes in DR (A) Schematic diagram of generation of Tgm2‐CreERT2 mice and the progeny of Tgm2CreERT2‐R26T/Gf/f mice. (B) Schematic diagram of hepatocyte fate tracing during 4‐week DDC injury in Tgm2CreERT2‐R26T/Gf/f mice. (C) Co‐staining of the CK19 and HNF4α with the GFP lineage label in the liver of normal or DDC‐fed mice after the injection of corn oil (n = 3/group). Scale bar, 10 μM. (D) Co‐staining of the hepatic stellate cell markers desmin, Col1a1, and αSMA with the GFP lineage label in normal adult mouse liver (n = 3/group). Scale bar, 20 μM. (E) Hepatic Tgm2 was measured in Tgm2CreERT2‐R26T/Gf/f mice after DDC injury by western blot (n = 3/group). (F) Masson's staining of liver samples after 4‐week DDC injury (n = 3/group). Scale bar, 100 μM. (G) Quantification of the percentage of collagen‐positive staining areas in Masson's staining (n = 4/group). (H) Hepatic expression levels of OPN and EpCAM were determined in Tgm2CreERT2‐R26T/Gf/f mice by RT‐qPCR analysis (n = 5/group). Comparisons between multiple groups were performed using ordinary one‐way ANOVA with Dunnett's multiple comparison test. Significant difference was presented at the levels of *p < 0.05, **p < 0.01, ***p < 0.001, and ****p < 0.0001. [file CPR-57-e13646-s008.tif]

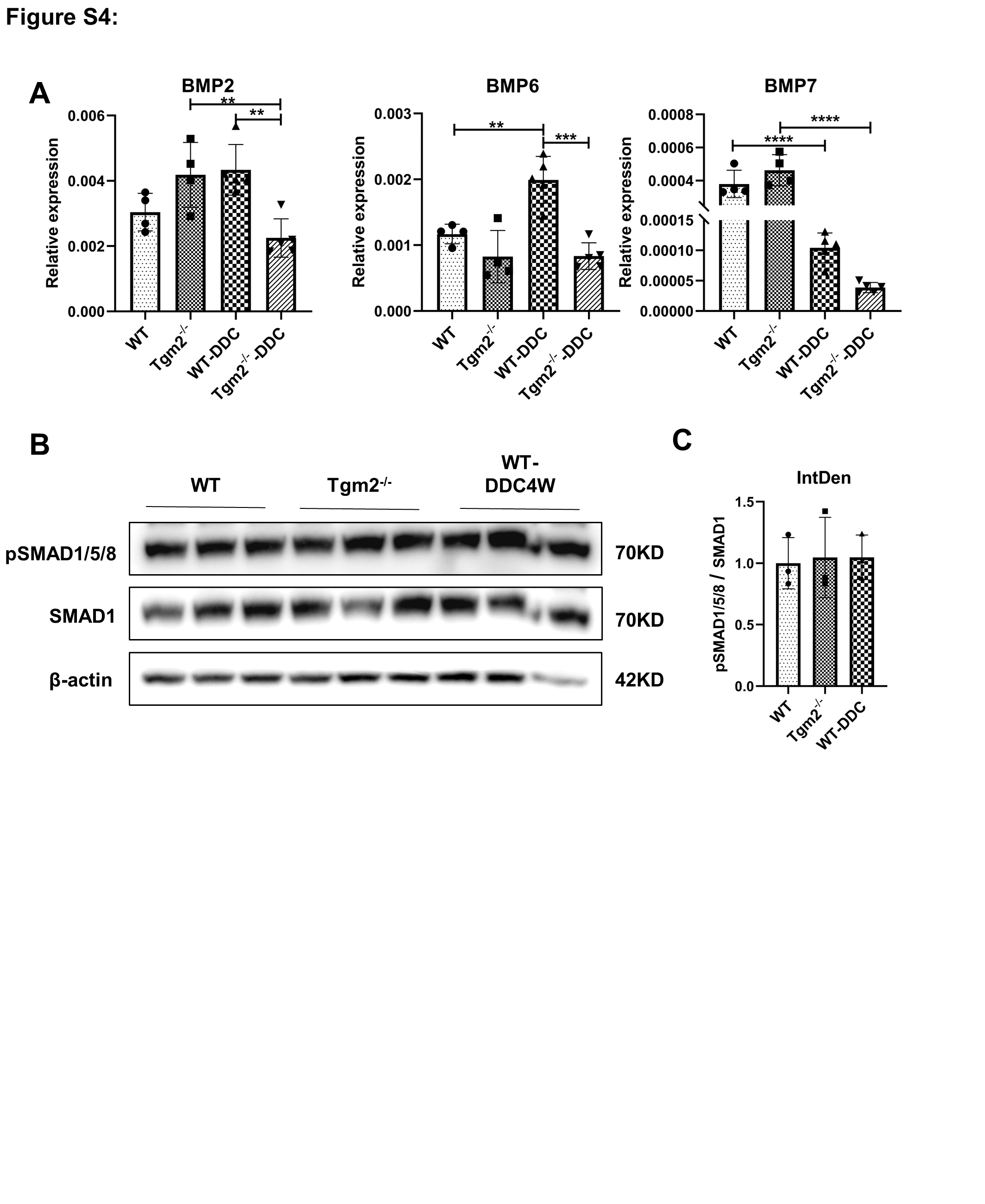

Supplement: Supplementary file 4 — Figure S4. There was no significant difference in BMP signalling between chow‐fed WT and Tgm2−/− mice (A) Hepatic expression levels of BMP2, BMP6, and BMP7 were determined by RT‐qPCR (n ≥ 4/group). (B) Western blot assay of pSMAD1/5/8, SMAD1, and β‐actin in liver extracts from WT and Tgm2−/− mice (n = 3/group). (C) Quantification of pSMAD1/5/8 measured by integrated density using ImageJ (n = 3/group). Comparisons between multiple groups were performed using ordinary one‐way ANOVA with Tukey's multiple comparison test. Comparisons between three groups were performed using ordinary one‐way ANOVA with Dunnett's multiple comparison test. Significant difference was presented at the levels of *p < 0.05, **p < 0.01, ***p < 0.001, and ****p < 0.0001. [file CPR-57-e13646-s007.tif]

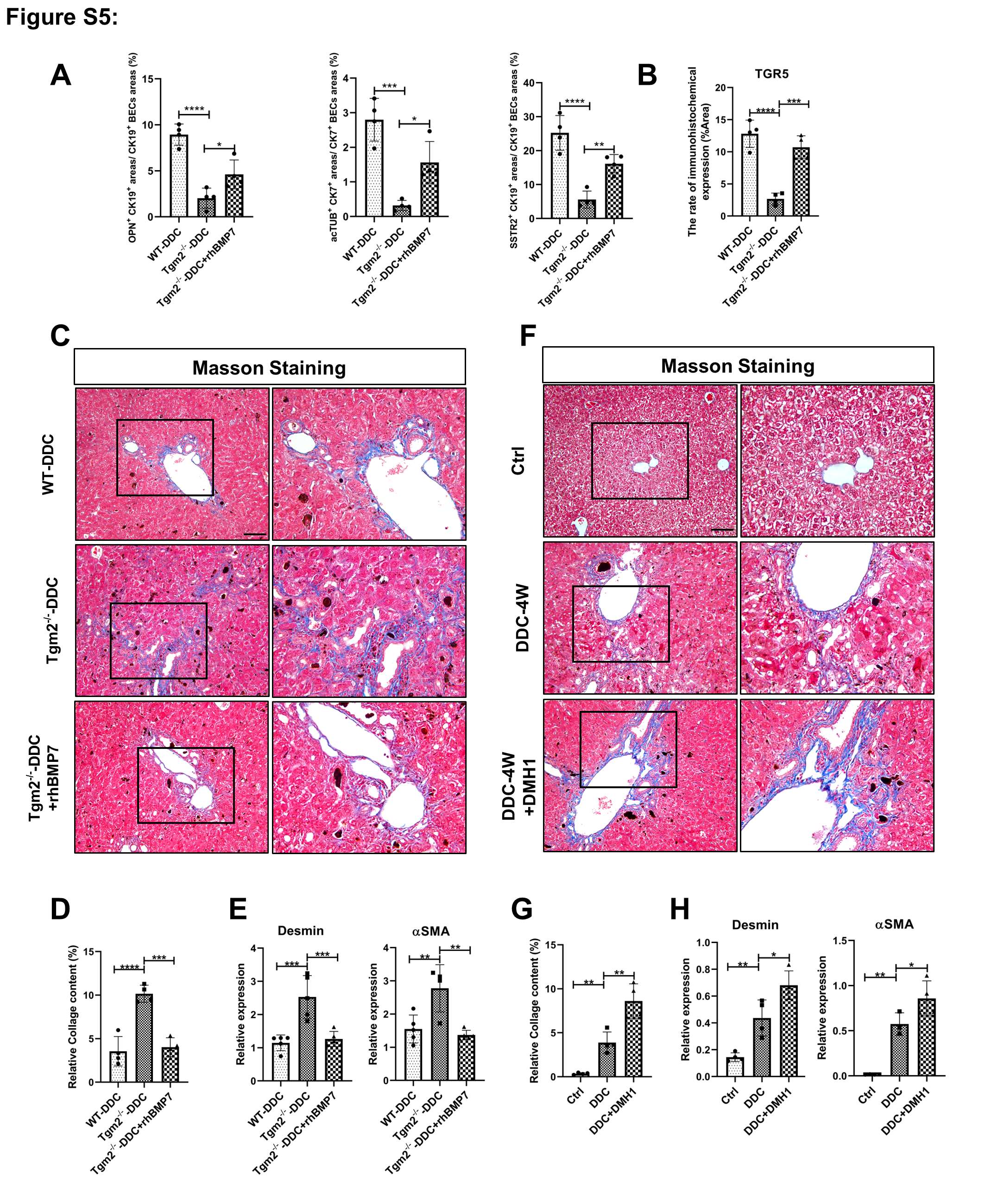

Supplement: Supplementary file 5 — Figure S5. Tgm2 signalling and BMP signalling influence the degree of liver fibrosis in DDC‐induced DR (A) The percentage of marker+ cells' (i.e., cells that stained positive for OPN, SSTR2, or acTUB) fluorescence intensity was determined in CK19+ or CK7+ BECs by fluorescence colocalization analysis after 4‐week DDC injury (n = 4/group). (B) Quantification of the percentage of TGR5+ area in immunohistochemical staining (n = 4/group). (C) Masson's staining in WT, Tgm2−/−, and Tgm2−/− + rhBMP7 groups (n = 4/group). Scale bar, 50 μM. (D) Quantification of the percentage of collagen‐positive staining areas in Masson's staining (n = 4/group). (E) Hepatic expression levels of desmin and αSMA were determined in WT, Tgm2−/−, and Tgm2−/− + rhBMP7 groups after 4‐week DDC injury (n = 4/group). (F) Masson's staining in Ctrl, DDC, and DDC + DMH1 groups (n = 4/group). Scale bar, 50 μM. (G) Quantification of the percentage of collagen‐positive staining areas in Masson's staining (n = 4/group). (H) Hepatic expression levels of desmin and αSMA were determined in Ctrl, DDC, and DDC + DMH1 groups after 4‐week DDC injury (n = 4/group). Comparisons between multiple groups were performed using ordinary one‐way ANOVA with Dunnett's multiple comparison test. Significant difference was presented at the levels of *p < 0.05, **p < 0.01, ***p < 0.001, and ****p < 0.0001. [file CPR-57-e13646-s002.tif]

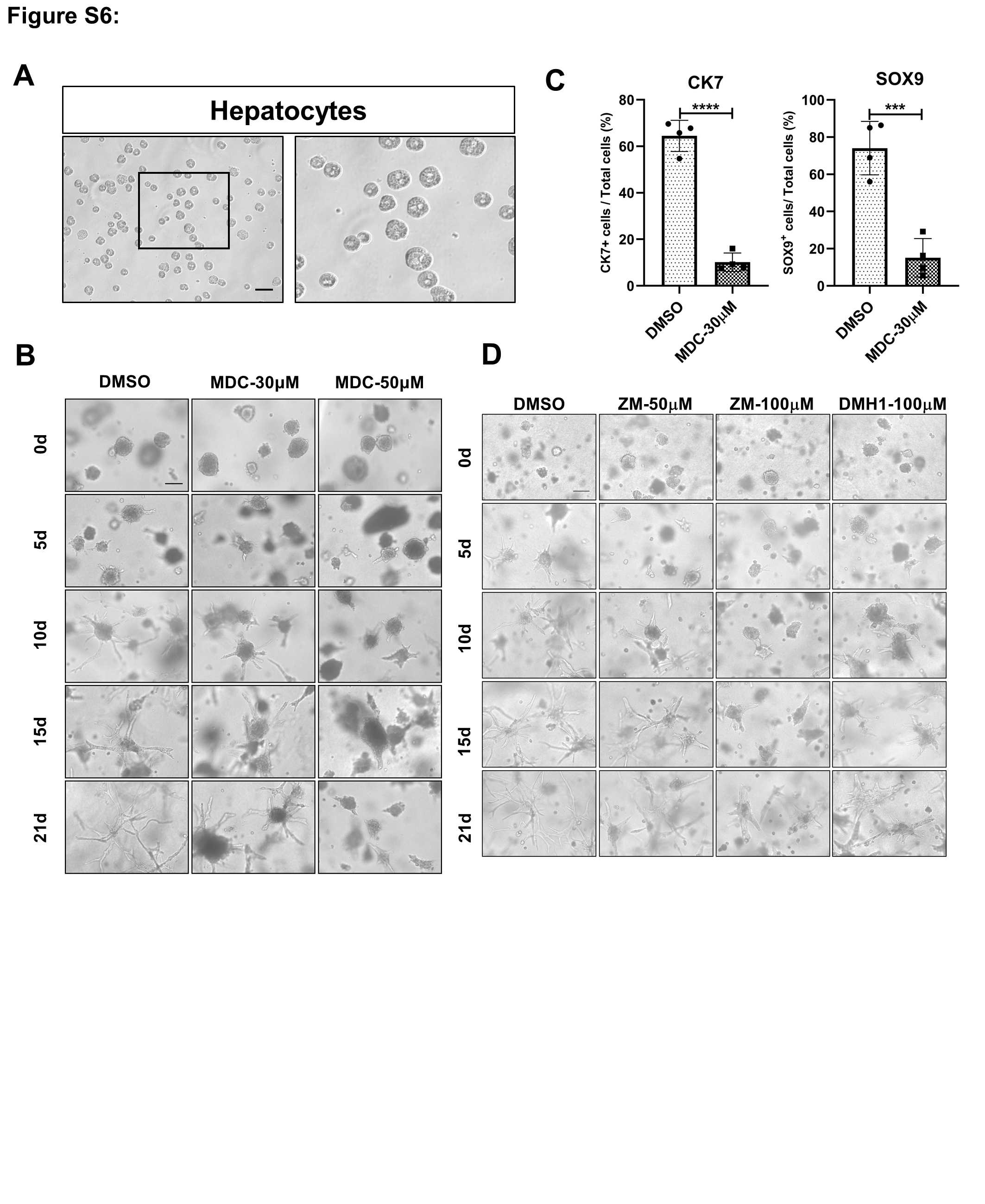

Supplement: Supplementary file 6 — Figure S6. Suppression of Tgm2 signalling or BMP signalling represses the transdifferentiation of hepatocytes, thus affecting the development and function of BECs in vitro (A) Phase‐contrast micrographs of freshly isolated hepatocytes. Scale bar, 50 μM. (B) Phase‐contrast micrographs of hepatocytic spheroids after being embedded in a collagen gel. The hepatocyte collagen gel culture was performed for 21 days in the absence or presence of MDC (30 or 50 μM) (n = 3/group). Scale bar, 50 μM. (C) The percentage of CK7+ or SOX9+ cells in total cells were measured in 3D collagen gel sections (n = 4/group). (D) Phase‐contrast micrographs of hepatocytic spheroids after 21‐day culture in a collagen gel in the absence or presence of ZM (50/100 μM) or DMH1 (100 μM) (n = 3/group). Comparisons between two groups were performed using two‐tailed Student's t‐test. *p < 0.05, **p < 0.01, ***p < 0.001, and ****p < 0.0001 represent 4 different levels of significant difference, respectively. [file CPR-57-e13646-s004.tif]
