# Supplementary material for: Deletion of Tgm2 suppresses BMP‐mediated hepatocyte‐to‐cholangiocyte metaplasia in ductular reaction
Source: Cell Prolif. 2024 Apr 16;57(10):e13646. doi: 10.1111/cpr.13646 (PMC11471396; doi:10.1111/cpr.13646)
Supplement: Supplementary file 7 — Table S1. Reagents and chemicals used for this study. Table S2. Antibodies used for this study. Table S3. List of primers used for genotyping and RT‐qPCR. [file CPR-57-e13646-s001.docx]

**Supplementary Table 1: Reagents and chemicals used for this study.**

| **Product** | **Company** | **Product Number** |
| --- | --- | --- |
| 0.1% 3,5-diethoxycarbonyl-1,4-dihydrocollidine (DDC) | Sigma | 137030 |
| DAPI | Abcam | ab104139 |
| Tamoxifen | Sigma | T5648 |
| Dorsomorphin homolog 1 (DMH-1) | MCE | HY-12273 |
| Dansylcadaverine (MDC) | MCE | HY-D1027 |
| Fluorescein Diacetate (FDA) | MCE | HY-D0719 |
| ZM39923 hydrochloride (ZM) | MCE | HY-12589 |
| Nicotinamide | MCE | HY-B0150 |
| Williams mediums' E | Gibco | A1217601 |
| Cellmatrix type I-A | Nitta | 637-00653 |
| Williams’ E medium (powder) | Caisson | WMP06 |
| Fetal bovine serum (FBS) | Gibco | 12484028 |
| Enhanced chemiluminescence | Beyotime | P0018S |
| Recombinant Human BMP-7 | Peprotech | 120-03P |
| Alkaline phosphatase Assay kit | Nanjing jiancheng | A059-2 |
| γ- Glutamyl transferase Assay Kit | Nanjing jiancheng | C017-2 |
| Alanine aminotransferase Assay Kit | Nanjing jiancheng | C009-2 |
| Aspartate aminotransferase Assay Kit | Nanjing jiancheng | C010-2 |
| Total Bilirubin Assay Kit | Nanjing jiancheng | C019-1-1 |
| Hematoxylin and Eosin Staining Kit | Beyotime | C0105S |
| Masson's Trichrome Stain Kit | Solarbio | G1340 |

**Supplementary Table 2: Antibodies used for this study**

| **Antibody** | **Company** | **Product number** | **RRID number** | **Application** | **Dilution** | **Host species** |
| --- | --- | --- | --- | --- | --- | --- |
| α-SMA | Servicebio | GB111364 |  | IF | 1:100 | Rabbit |
| Col1a1 | Servicebio | GB11022-3 |  | IF | 1:100 | Rabbit |
| Desmin | Servicebio | GB12081 |  | IF | 1:100 | Mouse |
| CK19 | Servicebio | GB12197 |  | IF | 1:100 | Mouse |
| OPN | Servicebio | GB11500 |  | IF | 1:50 | Rabbit |
| CK7 | Abcam | ab181598 | AB_2783822 | IF; IHC | 1:200 | Rabbit |
| acTUB | Sigma | T6793 | AB_477585 | IF | 1:200 | Mouse |
| SSTR2 | ABclonal | A15101 | AB_2761985 | IF | 1:300 | Rabbit |
| HNF4α | Abcam | ab41898 | AB_732976 | IF | 1:100 | Mouse |
| GFP | santa cruz | sc-9996 | AB_627695 | IF | 1:100 | Mouse |
| GFP | Proteintech | 50430 | AB_11042881 | IF | 1:100 | Rabbit |
| pSMAD1/5/8 | ABclonal | AP0850 | AB_2771539 | WB | 1:1000 | Rabbit |
| SMAD1 | Abcam | ab126761 | AB_11143606 | WB | 1:1000 | Rabbit |
| H3K4me3Q5ser | Sigma | ABE2580 |  | WB | 1:1000 | Rabbit |
| BMP7 | ABclonal | A0697 | AB_2757348 | WB | 1:1000 | Rabbit |
| β-actin | santa cruz | sc-58673 | AB_2223345 | WB | 1:2000 | Mouse |
| GAPDH | Proteintech | 60004-1-Ig | AB_2107436 | WB | 1:5000 | Mouse |
| Tgm2 | Abcam | ab2386 | AB_2287299 | WB | 1:1000 | Mouse |
| Sox9 | Millipore | AB5535 | AB_2239761 | IHC | 1:100 | Rabbit |
| TGR5 | ELK Biotechnology | ES4906 |  | IHC | 1:100 | Rabbit |
| EpCAM | Servicebio | GB1127 |  | IF | 1:150 | Rabbit |
| Biotin-conjugated Affinipure Goat Anti-Rabbit IgG(H+L) | Proteintech | SA00004-2 | AB_2890944 | WB，IHC | 1:1000；1：500 |  |
| Biotin-conjugated Affinipure Goat Anti-Mouse IgG(H+L) | Proteintech | SA00004-1 | AB_2890900 | WB | 1:1000 |  |
| Goat anti-Rabbit IgG (H+L) Cross-Adsorbed Secondary Antibody, Alexa Fluor 555 | Invitrogen | A-21428 | AB_2535844 | IF | 1:500 |  |
| Goat anti-Mouse IgG (H+L) Cross-Adsorbed Secondary Antibody, Alexa Fluor 488 | Invitrogen | A-11001 | AB_2534069 | IF | 1:500 |  |

**Supplementary Table 3 : List of primers used for genotyping and RT-qPCR.**

| **PCR Name** | **Primer sequence (5’-3’)** | **Amplification** |
| --- | --- | --- |
| 36B4-F | TGGAGACAAGGTGGGAGCC | RT-qPCR |
| 36B4-R | CACAGACAATGCCAGGACGC | RT-qPCR |
| BMP2-F | TCTTCCGGGAACAGATACAGG | RT-qPCR |
| BMP2-R | TGGTGTCCAATAGTCTGGTCA | RT-qPCR |
| BMP7-F | CCTGTCCATCTTAGGGTTGCC | RT-qPCR |
| BMP7-R | GGCCTTGTAGGGGTAGGAGA | RT-qPCR |
| BMP6-F | TCCCCACATCAACGACACCA | RT-qPCR |
| BMP6-R | TCCCCACCACACAGTCCTTG | RT-qPCR |
| BMP8a-F | CACCCTTCTCATCTGGATCG | RT-qPCR |
| BMP8a-R | CAGGAAGTAGGCACCGAGAG | RT-qPCR |
| OPN-F | ACGACCATGAGATTGGCAGT | RT-qPCR |
| OPN-R | CAGTCACTTTCACCGGGAGG | RT-qPCR |
| SSTR2-F | CTTGGCCATGCAGGTGGCGCTAGT | RT-qPCR |
| SSTR2-R | CAATGATGTCTTCCGCTCCGGATTGT | RT-qPCR |
| EpCAM-F | GCGGCTCAGAGAGACTGT | RT-qPCR |
| EpCAM-R | CCAAGCATTTAGACGCCAGTTT | RT-qPCR |
| ID1-F | GAACCGCAAAGTGAGCAAGG | RT-qPCR |
| ID1-R | GGAACACATGCCGCCTCA | RT-qPCR |
| Tgm2-F | GCTGGACCAACAGGACAATGT | RT-qPCR |
| Tgm2-R | CTCTAGGCTGAGACGGTACAG | RT-qPCR |
| CK19-F | GACCTAGCCAAGATCCTGAGT | RT-qPCR |
| CK19-R | TCAGCTCCTCAATCCGAGCA | RT-qPCR |
| Tgm2CreER2-common-F | CCAGGCCTGTCTCCTTACCTACCTT | Genotyping |
| Tgm2CreER2-WT-R | TCACAGCTAAGGGAACAGATGGGGA | Genotyping |
| Tgm2CreER2-MUT-R | CACATCCTCAGGTTCAGCAGGGAAC | Genotyping |
| mTmG-common-F | CTCTGCTGCCTCCTGGCTTCT | Genotyping |
| mTmG-WT-R | CGAGGCGGATCACAAGCAATA | Genotyping |
| mTmG-MUT-R | TCAATGGGCGGGGGTCGTT | Genotyping |
| Tgm2^-/-^-common-F | GTAAATCAAAAGCCGAACACCCTTG | Genotyping |
| Tgm2^-/-^-WT-R | ATCTCCAAATCACACCTCTCCAGG | Genotyping |
| Tgm2^-/-^-MUT-R | TCTGCTATGAGAAACGCTTACTTG | Genotyping |
| Bmp2-F | AGATCGAGCAAGTCCGCAAGCA | CHIP |
| Bmp2-R | CCTCCTCCCACCCACAACAAAT | CHIP |
| Bmp6-F | CGGTAACGGCATGGATTAAATAG | CHIP |
| Bmp6-R | TGCCTAGCGAGCGTAGGTTTCTA | CHIP |
| Bmp7-F | TGGCCTTTTAGGTTAGGTCTGAG | CHIP |
| Bmp7-R | CTCCTCATCAAGTGTCTATTGGGT | CHIP |
| Bmp8a-F | GGGGCTCATACCTGGCTACA | CHIP |
| Bmp8a-R | TACGGGTCCTACCGCTGCTC | CHIP |

**Supplementary Movie 1**

Video of 3D reconstruction of hepatocyte-to-biliary transdifferentiation in a collagen gel culture in vitro.
